# Molecular phenotypes of mitochondrial dysfunction in clinically non-manifesting heterozygous *PRKN* variant carriers

**DOI:** 10.1038/s41531-023-00499-9

**Published:** 2023-04-18

**Authors:** Maria Paulina Castelo Rueda, Alessandra Zanon, Valentina Gilmozzi, Alexandros A. Lavdas, Athina Raftopoulou, Sylvie Delcambre, Fabiola Del Greco M, Christine Klein, Anne Grünewald, Peter P. Pramstaller, Andrew A. Hicks, Irene Pichler

**Affiliations:** 1 Institute for Biomedicine, Eurac Research, Affiliated Institute of the University of Lübeck, Bolzano, Italy; 2 Department of Economics, University of Patras, Patras, Greece; 3 Luxembourg Centre for Systems Biomedicine, University of Luxembourg, Esche-sur-Alzette, Luxembourg; 4 Institute of Neurogenetics, University of Lübeck, Lübeck, Germany; 5 Department of Neurology, University Medical Center Schleswig-Holstein, Campus Lübeck, Lübeck, Germany

**Keywords:** Parkinson's disease, Mutation

## Abstract

Homozygous or compound heterozygous (biallelic) variants in *PRKN* are causal for PD with highly penetrant symptom expression, while the much more common heterozygous variants may predispose to PD with highly reduced penetrance, through altered mitochondrial function. In the presence of pathogenic heterozygous variants, it is therefore important to test for mitochondrial alteration in cells derived from variant carriers to establish potential presymptomatic molecular markers. We generated lymphoblasts (LCLs) and human induced pluripotent stem cell (hiPSC)-derived neurons from non-manifesting heterozygous *PRKN* variant carriers and tested them for mitochondrial functionality. In LCLs, we detected hyperactive mitochondrial respiration, and, although milder compared to a biallelic *PRKN*-PD patient, hiPSC-derived neurons of non-manifesting heterozygous variant carriers also displayed several phenotypes of altered mitochondrial function. Overall, we identified molecular phenotypes that might be used to monitor heterozygous *PRKN* variant carriers during the prodromal phase. Such markers might also be useful to identify individuals at greater risk of eventual disease development and for testing potential mitochondrial function-based neuroprotective therapies before neurodegeneration advances.

## Introduction

Parkinson´s disease (PD) is a neurodegenerative movement disorder, with both genetic and environmental factors playing an important role in disease onset, and age being the biggest risk factor for developing the disease^1,2^. PD is clinically characterized by cardinal motor manifestations^3,4^, which are often preceded by non-motor symptoms for years or even decades^2^. Even though most PD cases are classified as sporadic, a minority of cases (~10%) have a clear monogenic, autosomal dominant, or recessive, inheritance pattern^5^. To date, rare variants in more than 20 genes have been reported to cause PD, although only about half of them are unequivocally linked to PD^5^.

Impairment of mitochondrial function has been highlighted as a key mechanism of this pathology, and several genes implicated in familial forms of PD, including *PRKN (Parkin), PINK1, PARK7 (DJ-1)*, and *SNCA* (alpha-synuclein) are involved in the regulation of mitochondrial function^6^. *PRKN*-linked PD is the most common form of early-onset PD across all ethnic groups, representing about 10–20% of cases with the age of onset (AAO) from 40–50 years^7,8^ and up to 42.2% of cases with an AAO ≤20 years^9^. Homozygous or compound heterozygous (biallelic) variants in *PRKN* are causal for PD with highly penetrant symptom expression and early AAO, but may manifest with late onset in a subset of cases^8,10^. The much more common heterozygous variants in this gene may predispose to PD with highly reduced penetrance^11,12^. However, the role of heterozygous *PRKN* variants as causative or susceptibility factors for PD is still under vivid debate: two studies have found an increased risk for PD in carriers of heterozygous *PRKN* variants as compared to controls^13,14^, while two other studies with almost complete genotyping of *PRKN* for copy number and single nucleotide polymorphisms found that heterozygous *PRKN* variants do not contribute to overall disease risk^15,16^. In the latter papers, however, there was no possibility to assess subtle, subclinical phenotypes that might be present in the carriers.

Heterozygous variant carriers present an extremely variable phenotype expressivity ranging from no clinical signs to subtle motor signs, which do not allow for a definitive clinical diagnosis of PD, to overt symptoms of PD^10,17,18^. Importantly, a dopaminergic deficit has been clearly observed by positron emission tomography in studies with asymptomatic heterozygous *PRKN* variant carriers^19–21^, with a mean annual reduction in the putamen and caudate of 0.56 and 0.62%, respectively, compared to 0.5 and 2% in biallelic *PRKN* mutation carriers^22^.

Pathogenic variants in *PRKN* can affect Parkin function through different mechanisms impairing its activity, stopping the translation of a functional protein, rendering the protein insoluble and enhancing aggregation, hampering protein folding and stability, and/or affecting its ability to bind to cofactors and substrates^23^. Overall, it is a complex protein exerting numerous effects, which undeniably contribute to cellular homeostasis and survival. In particular, Parkin deficiency was proven to disrupt mitochondrial performance, demonstrating the crucial role of these organelles in the onset of *PRKN*-linked disease^24,25^. Parkin protein is an E3 ubiquitin ligase, which is localized in the cytoplasm and translocates to the mitochondria upon specific stimulation^26,27^. It ubiquitinates many substrate proteins, including itself, for proteasomal degradation or signaling processes^28,29^. Following mitochondrial membrane depolarization, Parkin is recruited to mitochondria and activated by PINK1, resulting in the ubiquitination of outer mitochondrial membrane proteins, including Mitofusin-1 and Mitofusin-2, which are essential for mitochondrial fusion^30^. Concurrently with proteasomal degradation of mitochondrial substrate proteins, components of the autophagy machinery are recruited, leading to the selective autophagic removal of the damaged organelle, a process known as mitophagy^31,32^. Additionally, Parkin is involved in the activation of mitochondrial biogenesis through ubiquitination and subsequent degradation of PARIS (parkin interacting substrate), a transcriptional repressor of the master regulator of mitochondrial biogenesis, PGC1-α (peroxisome proliferator-activated receptor gamma coactivator 1α)^33^ or via metabolic modulation of the Sirtuin1/PGC1-α pathway^34^. Furthermore, Parkin is involved in mitochondrial DNA (mtDNA) maintenance^34,35^. Interestingly, after crossing the Mutator mouse, which possesses a proofreading-deficient form of POLG, the polymerase responsible for mtDNA replication, resulting in accumulation of mtDNA deletions, with a *PRKN* knock-out mouse, which both by themselves do not show neurodegeneration, the resulting double knockout displays obvious mitochondrial dysfunction and PD pathology. This was linked to a greater predicted pathogenicity score for mtDNA variants^35^. Moreover, in iPSC-derived POLG-mutant neurons deficient in Parkin, mtDNA dyshomeostasis coincided with the release of mtDNA molecules into the extracellular space, where they can propagate inflammation^34^.

Considering that the frequency of heterozygous *PRKN* variant carriers in the population is up to 4%^11,36^, and that some of these carriers may eventually develop the disease, it is important to further elucidate the role of pathogenic heterozygous variants in this recessive gene, by testing the causal link between the heterozygous variants and expressivity of molecular phenotypes. Therefore, in this study, we sought to evaluate alterations of mitochondrial integrity, including disparities at the level of mtDNA and mitochondrial function, in blood and cellular models of non-manifesting heterozygous *PRKN* variant carriers.

## Results

### Enhanced mitochondrial respiration in intact lymphoblasts of heterozygous *PRKN* exon 7 deletion carriers

**Fig. 1:**
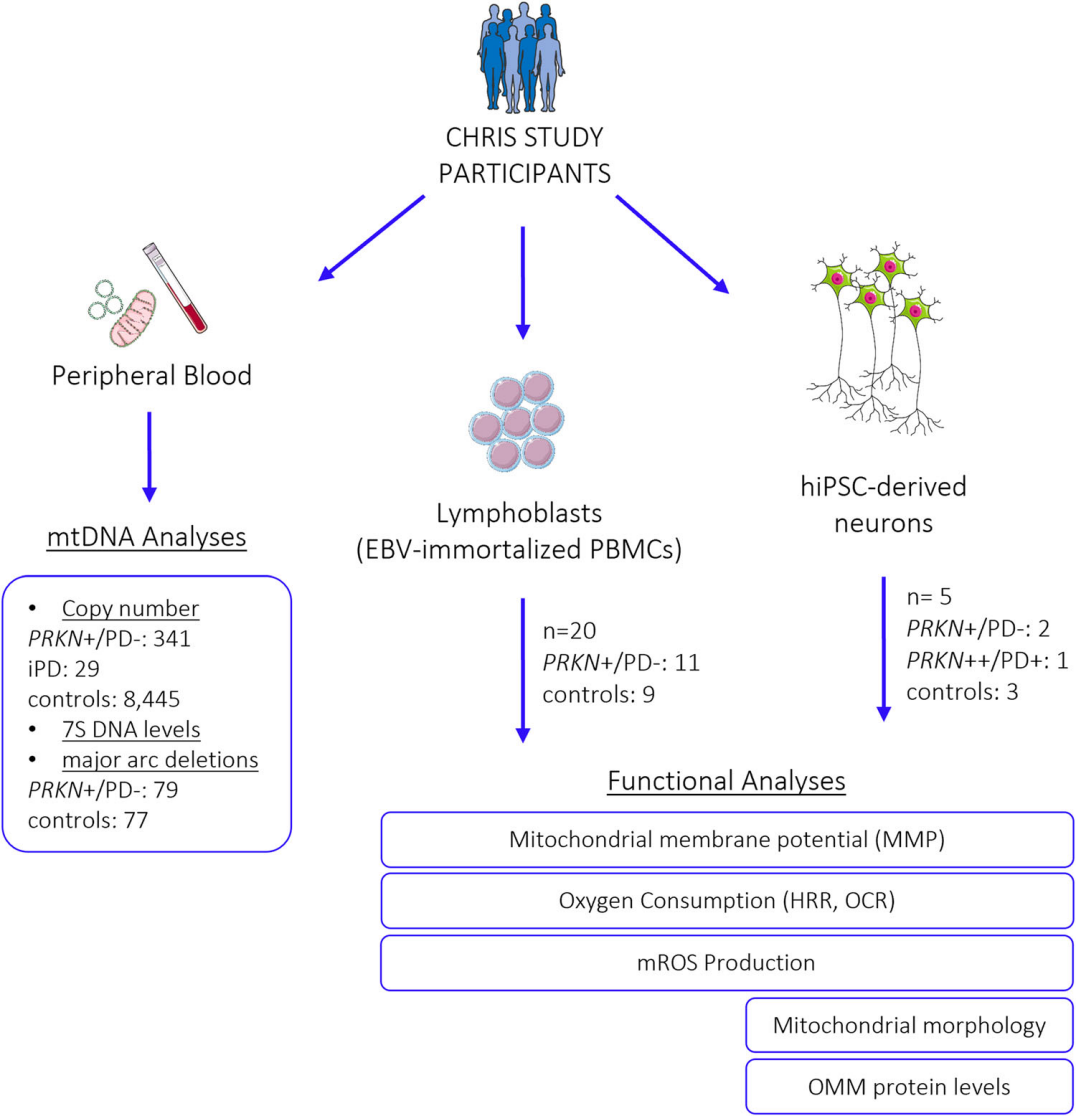
Experimental design. Flow chart indicating study participants, biospecimens, and performed experiments. PBMCs peripheral blood mononuclear cells, EBV Epstein Barr Virus, mtDNA mitochondrial DNA, LCLs lymphoblastoid cell lines, MMP mitochondrial membrane potential, mROS mitochondrial reactive oxygen species, OMM outer mitochondrial membrane. *PRKN*+/PD−: individuals carrying a heterozygous variant in *PRKN*; *PRKN*++/PD+: PD patient with a homozygous variant in *PRKN*; iPD: patient with idiopathic PD. Details on study participants are available in Supplementary Table 1. This figure was created using elements from Servier Medical Art (https://smart.servier.com), which is licensed under a Creative Commons Attribution 3.0 Unported Generic License (https://creativecommons.org/licenses/by/3.0/).

**Fig. 2:**
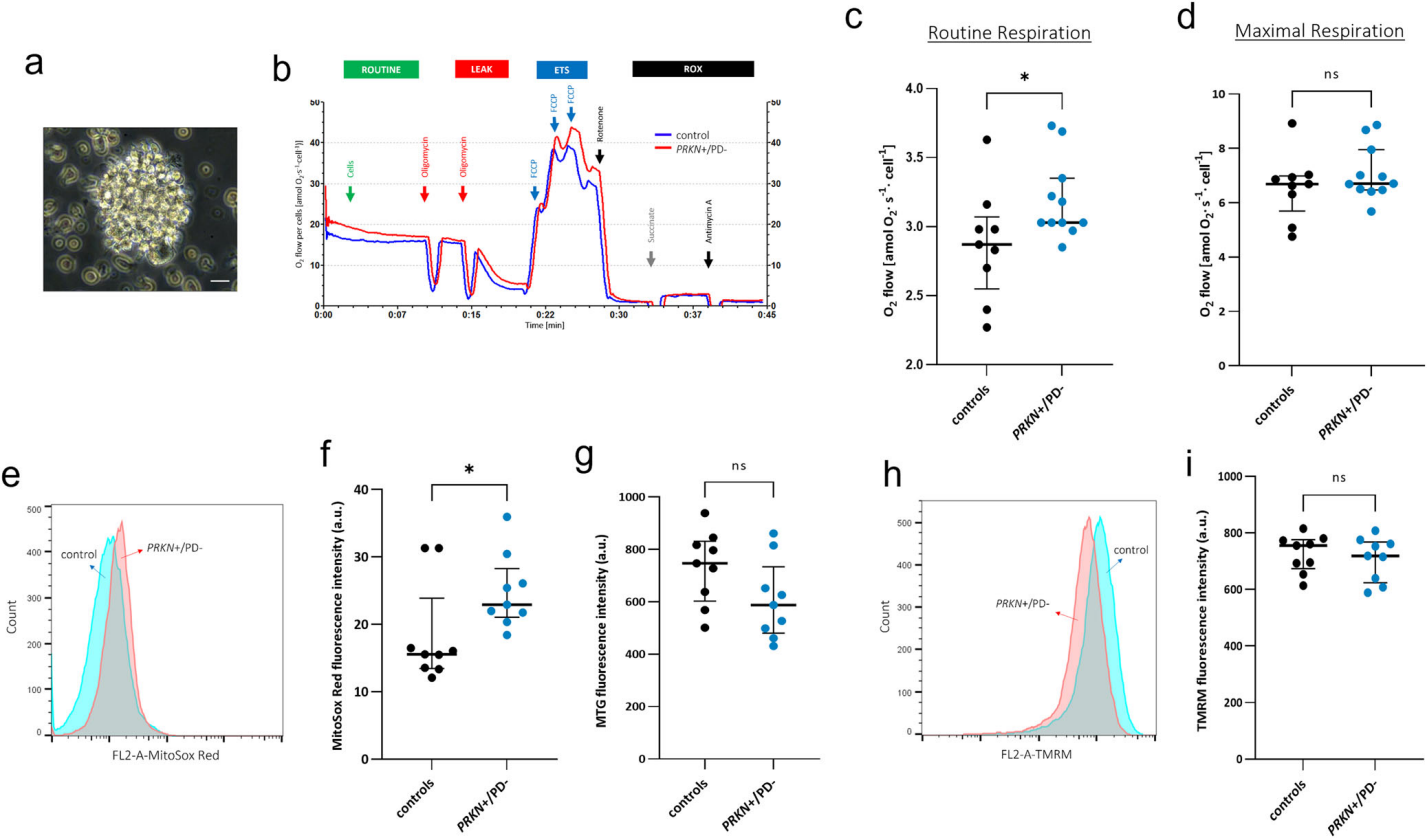
Altered mitochondrial function in LCLs of heterozygous *PRKN* variant carriers. **a** Visualization of LCLs by phase contrast inverted microscope: cell clump showing typical rosette morphology, scale bar: 25 µm. **b** Representative traces of real-time high-resolution respirometry using an intact substrate-uncoupler-inhibitor titration (SUIT) protocol. The oxygen flow per cells (y-axis) is represented as a function of time (x-axis). Arrows indicate the addition of cells and titrations of substrates and inhibitors, which comprised: the addition of 5 × 10^−6^ cells in each chamber; oligomycin (5 nM each step); FCCP (0.5 µM each step); rotenone (0.5 µM); succinate (10 mM); antimycin A (2.5 µM). Representative trace for one control (blue trace) and one heterozygous exon 7 deletion carrier (red trace). Oxygen fluxes were normalized for cell count and citrate synthase activity. ETS electron transfer system; ROX residual oxygen consumption. Group-wise comparison of routine respiration (**c**) and maximal respiration (**d**) in LCLs from exon 7 heterozygous deletion carriers (*PRKN*+/PD−, *n* = 11) and controls (*n* = 9), characterized by mitochondrial oxygen flow per cell. Each line was measured in *n* ≥ 3 independent assays. Data were presented as scatter plots showing a median with an interquartile range. Statistical analysis was carried out by using the Mann–Whitney *U*-test. (*) indicates significant group differences with an alpha level of 0.05. *p* = 0.023; ns not significant. **e** Representative flow cytometry histograms of MitoSox Red fluorescence in LCLs (20,000 cells). **f** Group-wise comparison of mROS production in LCLs of heterozygous variant carriers (*PRKN*+/PD−, *n* = 9) and controls (*n* = 9). Mitotracker green (MTG) was used to normalize the MitoSox Red signal to mitochondrial content. **g** Group-wise comparison of mitochondrial content (MTG signal) in LCLs of heterozygous variant carriers (*PRKN*+/PD−, *n* = 9) and controls (*n* = 9). *n* ≥ 3 independent experiments. Results are presented as scatter plots showing the median with interquartile range; statistical analysis was carried out by using Mann–Whitney *U*-test; *p* = 0.031. a.u. arbitrary units. **h** Representative flow cytometry histograms of TMRM fluorescence in LCLs (20,000 cells). **i** Group-wise comparison of MMP in LCLs from heterozygous variant carriers (*PRKN*+/PD–, *n* = 9) and controls (*n* = 9). *n* ≥ 3 independent experiments. Results are presented as scatter plots showing the median with interquartile range; statistical analysis was carried out with Mann–Whitney *U*-test; *p* = 0.604. a.u. arbitrary units. ns not significant.

From 20 individuals (11 carriers of a heterozygous *PRKN* exon 7 deletion and 9 controls) (Fig. 1 and Supplementary Table 1), lymphoblastoid cell lines (LCLs) (Fig. 2a) were derived, and protein levels of Parkin were estimated using an antibody able to recognize only Parkin with an intact C-terminus (Supplementary Fig. 1a). In heterozygous mutation carriers, protein levels were ∼50% of those detected in controls. Oxygen consumption was measured by means of high-resolution respirometry; representative traces of oxygen consumption in intact LCLs illustrate the applied multiple substrate-uncoupler-inhibitor titration (SUIT) protocol (Fig. 2b). Under physiological conditions of non-permeabilized LCLs, individuals carrying a heterozygous exon 7 deletion in *PRKN* present a significantly increased oxygen consumption, identified in the routine respiratory state (*p* = 0.023, 3.03 vs 2.87) (Fig. 2c), suggesting hyperactive mitochondrial respiration in LCLs derived from heterozygous variant carriers. For subsequent respiratory states, including proton leak and maximal respiratory capacity (Fig. 2d and Supplementary Table 2), group differences were not statistically significant. Accordingly, ATP turnover, calculated by the decrease in oxygen consumption upon injection of the ATP synthase inhibitor oligomycin, suggests an increased ATP production in the LCLs of the heterozygous variant carriers. These data indicate hyperactive mitochondrial respiration in blood-derived cells of carriers of a pathogenic heterozygous *PRKN* variant.

### Lymphoblasts of heterozygous exon 7 deletion carriers exhibit elevated mROS production and unaltered mitochondrial membrane potential

To measure mitochondrial ROS (mROS) production, LCLs of heterozygous exon 7 deletion carriers and controls were stained with MitoSox Red dye, and fluorescence intensity was measured by flow cytometry. As depicted in Fig. 2e, f, the quantification of the MitoSox Red signal showed an increase of mROS in the LCLs of heterozygous variant carriers, compared to the control group (*p* = 0.031, 22.90 vs. 15.55, respectively).

We further evaluated the mitochondrial membrane potential (MMP), which is considered an important parameter of mitochondrial integrity^37^, by using the cell-permeant dye TMRM (tetramethylrhodamine methyl ester). Flow cytometry analysis of the fluorescence intensity showed no significant difference in the LCLs of the carriers compared to the control group (*p* = 0.604, 718.7 vs. 755.7) (Fig. 2h, i). Collectively, increased mROS production, but unchanged MMP, are in line with the enhanced mitochondrial respiratory activity and enhanced electron transport observed in the heterozygous carrier group.

### Human iPSC-derived neurons of heterozygous variant carriers suggest a reduced oxygen consumption rate

We further investigated mitochondrial phenotypes in DA neurons of heterozygous exon 7 deletion carriers as a second cell type, which might be more susceptible to changes induced by a *PRKN* variant, since they might rely predominantly on mitochondrial oxidative metabolism^38–40^. We reprogrammed peripheral blood mononuclear cells (PBMCs) of four individuals (two carriers of a heterozygous exon 7 deletion and two controls; for three of them, we also generated LCLs) to human induced pluripotent stem cells (hiPSCs)^41^ (Fig. 1 and Supplementary Table 1) and differentiated them to DA neurons (Supplementary Fig. 1b, c). We also included a homozygous *PRKN*-PD hiPSC line (exon 3 deletion or 1-bp deletion in exon 9) and an additional control line in all experiments (Supplementary Table 1). Protein levels of Parkin were estimated using an antibody able to recognize only Parkin with an intact C-terminus. In hiPSC-derived DA neurons from heterozygous mutation carriers, protein levels were ∼50% of those detected in controls, and no Parkin protein was detectable in the homozygous *PRKN* mutation carrier lacking the C-terminal part of Parkin (Supplementary Fig. 1b).

**Fig. 3:**
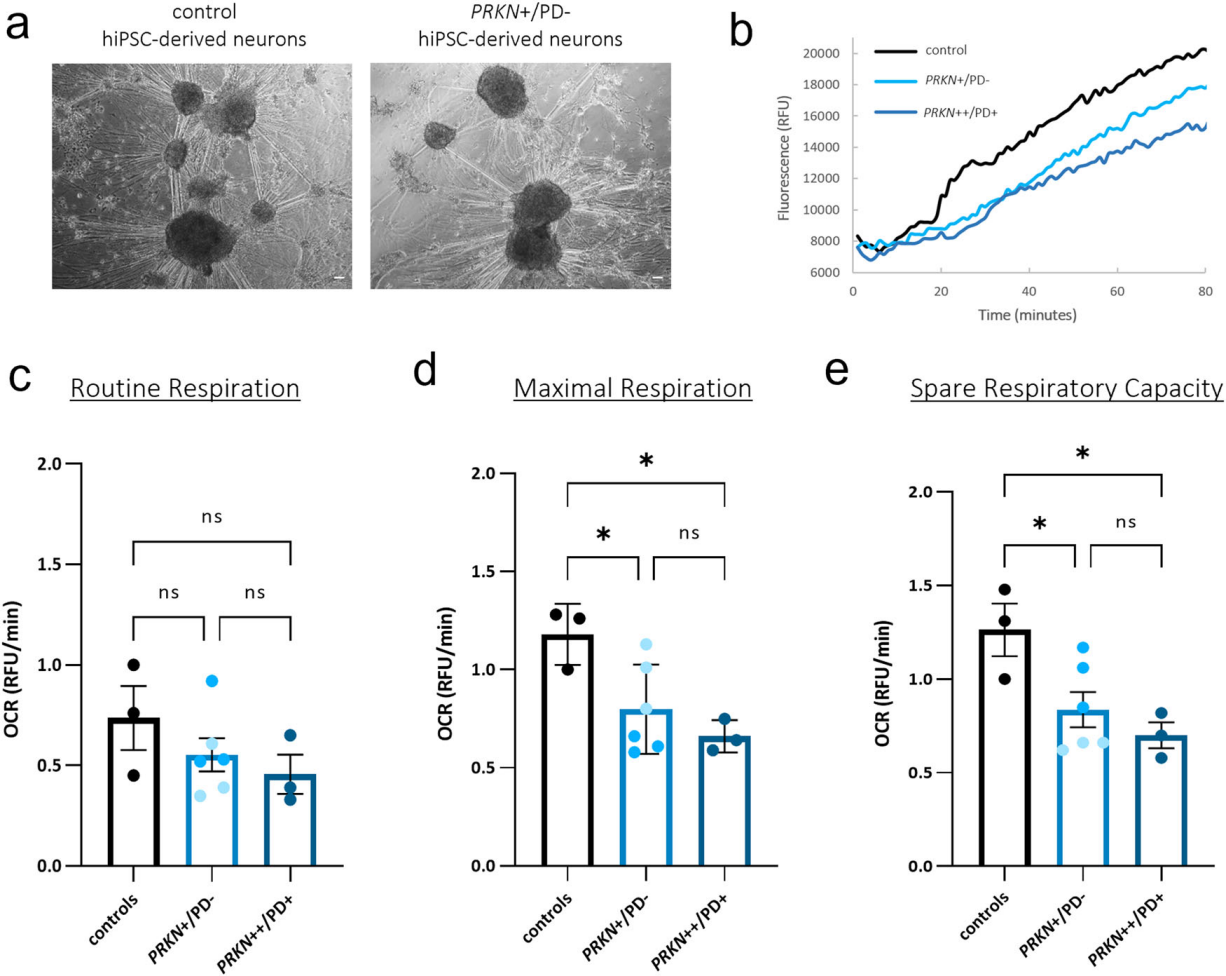
Reduced oxygen consumption rates in hiPSC-derived neurons of heterozygous *PRKN* variant carriers. **a** Representative images visualized by phase contrast inverted microscope of hiPSC-derived neurons used for OCR assessment at day 34 of differentiation. Scale bar: 100 µm. **b** Representative maximal respiration curves of the fluorescence signal, generated by the oxygen probe after the addition of 2.5 µM CCCP. Curves reflect dissolved oxygen in the culture medium of hiPSC-derived neurons of a heterozygous *PRKN* variant carrier (*PRKN*+/PD−), a control, and a *PRKN*-PD patient with a homozygous variant (*PRKN*++/PD+). Relative quantification of the respirometry parameters: **c** routine respiration, **d** maximal respiration measured after the addition of 2.5 µM CCCP, and **e** spare respiratory capacity (maximal respiration—routine respiration) in hiPSC-derived neurons of a PD patient with a homozygous variant (*PRKN*++/PD+), in the carrier group (*PRKN*+/PD−) comprised of two heterozygous variant carriers (*PRKN*+/PD− 1 and *PRKN*+/PD− 2, respectively color coded) and the control group (including the mean values of two non-carrier controls from the same population and an additional control line). *n* = 3 experiments on 3 independent differentiations (three wells per respiratory parameter for each differentiation). Error bars represent the mean ± SEM of three data points (one for each differentiation) for each experimental group. Each data point was calculated with the values of one PD patient, two heterozygous variant carriers, and three controls for each differentiation. *p* values were determined using one-way ANOVA followed by Tukey’s post hoc test to correct for multiple comparisons. *p ≤ 0.05, ns not significant, RFU relative fluorescence units.

To measure oxidative phosphorylation activity in hiPSC-derived neurons (Fig. 3a), we quantified extracellular oxygen consumption rate (OCR) under basal conditions (routine respiration) and upon treatment with the protonophore CCCP (carbonyl cyanide 4-(trifluoromethoxy) phenylhydrazone), leading to mitochondrial uncoupling (Fig. 3b). The two non-manifesting individuals carrying a heterozygous *PRKN* variant, and the homozygous *PRKN*-PD patient showed a trend towards a decreased basal OCR level compared to the control group (controls 0.73 ± 0.1 vs. 0.55 ± 0.1 vs. 0.45 ± 0.17; *p* = 0.48, *p* = 0.29) (Fig. 3c). When challenging the neurons with CCCP to induce maximal respiration, both heterozygous variant carriers and the homozygous *PRKN*-PD line revealed a significant decrease of oxygen consumption (controls 1.18 ± 0.13 vs. 0.79 ± 0.1 vs. 0.66 ± 0.15; *p* = 0.044, *p* = 0.02, respectively) (Fig. 3d). Spare respiratory capacity indicates the mitochondrial capability to respond to a chronic mitochondrial insult, which might be the presence of a single *PRKN* variant. This parameter was significantly reduced for the homozygous *PRKN*-PD line (1.26 ± 0.15 vs. 0.70 ± 0.17; *p* = 0.026) but also for the heterozygous *PRKN* variant lines (1.26 ± 0.15 vs. 0.83 ± 0.15; *p* = 0.049) (Fig. 3e). The decrease in OCR levels at maximal respiration as well as for the spare respiratory capacity was seemingly more pronounced in the PD patient line with homozygous *PRKN* variants as compared to the heterozygous variant carriers, although the difference between these two groups was not significant (*p* = 0.57, *p* = 0.65, respectively).

### Oxidative stress levels in hiPSC-derived neurons of heterozygous variant carriers

**Fig. 4:**
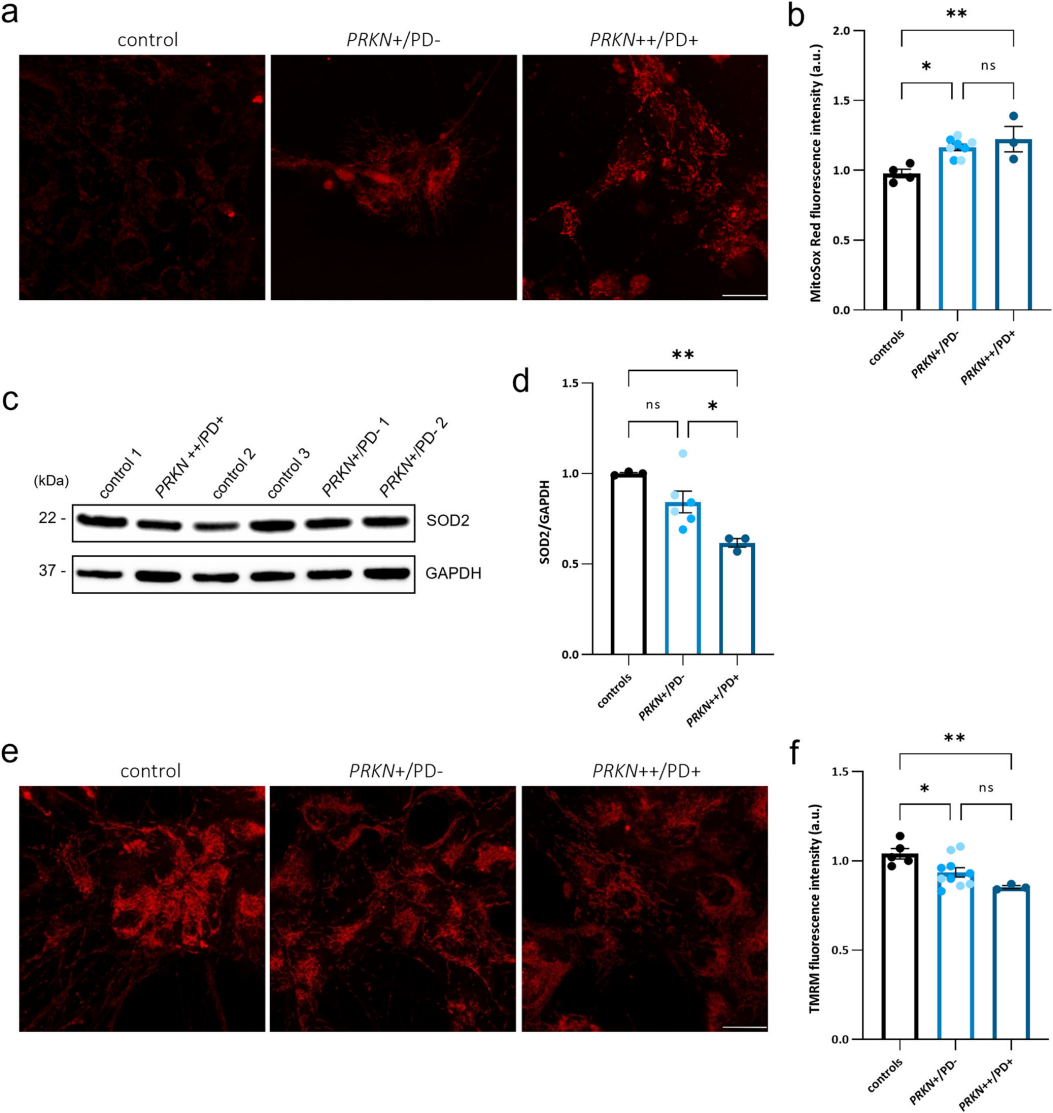
Increased mitochondrial ROS and reduced membrane potential in hiPSC-derived neurons of heterozygous *PRKN* variant carriers. **a** Confocal live imaging using MitoSox Red staining in hiPSC-derived neurons at day 40 of differentiation. Scale bar: 25 µm. **b** Quantification of mean fluorescence intensity of MitoSox Red in hiPSC-derived neurons of a PD patient line with a homozygous variant (*PRKN*++/PD+), the carrier group (*PRKN*+/PD−) comprised of two heterozygous variant carriers (*PRKN*+/PD− 1 and *PRKN*+/PD− 2, respectively color coded), and the control group (including the mean values of three control lines per experiment). *n* = 4 independent experiments. **c**, **d** Representative Western blot and subsequent densitometric analysis showing SOD2 protein expression in neurons of the PD patient with a homozygous variant (*PRKN*++/PD+), the carrier group (*PRKN*+/PD−) comprising two heterozygous carriers (*PRKN*+/PD− 1 and *PRKN*+/PD− 2, respectively color coded), and the control group (representing the mean values of three control lines per experiment). SOD2 levels were analyzed with the indicated anti-SOD2 antibody, and GAPDH was used as a loading control. Molecular mass markers are in kilodaltons (kDa). *n* = 3 independent experiments. **e** Confocal live imaging of hiPSC-derived neurons at day 40 of differentiation, indicating mitochondria stained with TMRM. Scale bar: 25 µm. **f** Quantification of mean fluorescence intensity of TMRM in hiPSC-derived neurons of a PD patient line with homozygous variants (*PRKN*++/PD+), the carrier group (*PRKN*+/PD−) comprised of the two heterozygous carriers (*PRKN*+/PD− 1 and *PRKN*+/PD− 2, respectively color coded) and the control group (representing the mean values of three control lines per experiment). *n* = 5 independent experiments. The mean fluorescence intensity of MitoSox Red and TMRM was analyzed in thresholded images, using the Image J software. Cell imaging and quantification were performed in at least 50 cells per sample for each experiment. In each case, data represent the mean ± SEM from the independent differentiations. *p* values were determined using one-way ANOVA followed by Tukey’s post hoc test to correct for multiple comparisons. *p ≤ 0.05, **p ≤ 0.01, ns not significant, a.u. arbitrary units.

To explore oxidative stress in hiPSC-derived neurons of individuals carrying a heterozygous variant in *PRKN*, we measured mitochondrial superoxide (mROS) by staining with the fluorogenic dye MitoSox Red. As depicted in Fig. 4a, neurons obtained from the heterozygous variant carriers as well as from the PD patient carrying a homozygous *PRKN* variant, displayed MitoSox-positive mitochondria with a significantly higher mean fluorescence intensity as compared to the control neurons (0.97 ± 0.05 vs. 1.16 ± 0.05 vs. 1.22 ± 0.06; *p* = 0.010, *p* = 0.007, respectively) (Fig. 4b). When comparing mROS levels between the heterozygous variant carriers and the PD patient, no statistically significant difference was detected (*p* = 0.594. 1.16 ± 0.05 vs. 1.22 ± 0.06, respectively) but the direction of the effect observed for the respiration experiments was confirmed. This suggests increased oxidative stress levels in the hiPSC-derived neurons carrying a heterozygous *PRKN* variant compared to the controls, similar to the phenotype observed in the homozygous *PRKN*-PD patient.

mROS levels are partly regulated by the mitochondrial antioxidant enzyme superoxide dismutase 2 (SOD2, MnSOD)^42,43^. To investigate the regulation of mROS in our lines, we quantified the protein levels of SOD2 by means of Western blot (Fig. 4c). Interestingly, SOD2 levels in the patient carrying a homozygous *PRKN* variant were significantly decreased when compared to the control group (1.00 ± 0.07 vs. 0.61 ± 0.09, *p* = 0.005) (Fig. 4d). For the individuals carrying a heterozygous variant, a trend towards reduction was observed compared to the control group (0.84 ± 0.07 vs. 1.00 ± 0.07; *p* = 0.169), which might suggest deficiencies in the defense mechanism against oxidative stress in individuals either lacking Parkin or showing reduced Parkin levels.

### Mitochondrial membrane potential alterations in hiPSC-derived neurons of heterozygous variant carriers

MMP was assessed using the cell-permeant dye TMRM and live imaging confocal microscopy (Fig. 4e). Mean fluorescence intensity analysis revealed a significant reduction of MMP for the heterozygous *PRKN* variant carriers (*p* = 0.049) as well as for the homozygous *PRKN*-PD patient neurons (*p* = 0.008), compared to the control group (1.04 ± 0.04 vs. 0.93 ± 0.03 vs. 0.85 ± 0.05) (Fig. 4f). Taken together, these results indicate an altered mitochondrial functionality in the presence of disease caused by homozygous *PRKN* variants, which is detectable also in non-manifesting carriers of a heterozygous *PRKN* variant.

### Mitochondrial network morphology is unaffected in hiPSC-derived neurons of heterozygous variant carriers in *PRKN*

**Fig. 5:**
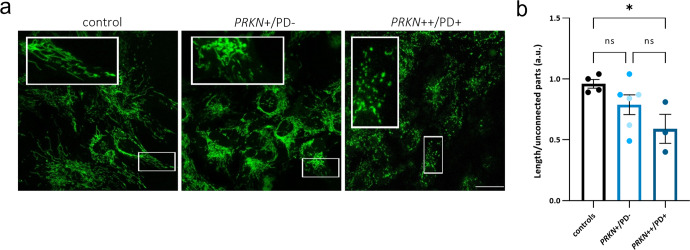
Mitochondrial network morphology is unaltered in hiPSC-derived neurons of heterozygous *PRKN* variant carriers. **a** Confocal live imaging of hiPSC-derived neurons at day 50 of differentiation, stained with MTG. Scale bar: 25 µm. **b** Mitochondrial fragmentation measurements in hiPSC-derived neurons of a PD patient line with homozygous variants (*PRKN*++/PD+), the carriers group (*PRKN*+/PD−) comprising two heterozygous carriers (*PRKN*+/PD− 1 and *PRKN*+/PD− 2, respectively color coded), and the control group (representing the mean values of three control lines per experiment). *n* = 4 independent experiments. Cell imaging and calculations were performed in at least 50 cells per sample for each experiment. Mitochondrial fragmentation was calculated with the filament function in Imaris Software: for each field, the total length of the modeled mitochondrial network was calculated and divided by the number of unconnected parts. Error bars represent the mean ± SEM. *p* values were determined using one-way ANOVA followed by Tukey’s post hoc test to correct for multiple comparisons. *p ≤ 0.05. a.u. arbitrary units, ns not significant.

Considering the functional mitochondrial impairments observed so far in the neuronal model, we performed a morphological analysis of the mitochondrial network by staining with the fluorescent dye Mitotracker green (MTG), which specifically localizes to mitochondria regardless of their membrane potential (Fig. 5a). hiPSC-derived neurons from the patient carrying a homozygous *PRKN* variant revealed an increased mitochondrial fragmentation (quantified as the total length of the modeled mitochondrial network divided by the number of unconnected parts), when compared to the control group (0.96 ± 0.11 vs. 0.59 ± 0.13; *p* = 0.044), while heterozygous *PRKN* variant carriers showed a trend towards increased mitochondrial fragmentation without a statistically significant group change (0.96 ± 0.11 vs. 0.78 ± 0.11; *p* = 0.313) (Fig. 5b). The direction of the effect of these data coincides with the altered mitochondrial functionality observed in hiPSC-derived neurons of diseased homozygous and non-manifesting single variant carriers in *PRKN*.

### Abundance of outer mitochondrial membrane proteins in hiPSC-derived neurons of heterozygous *PRKN* variant carriers

**Fig. 6:**
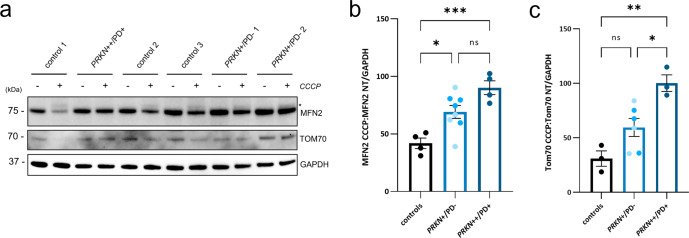
Elevated steady-state levels of OMM proteins upon stress in hiPSC-derived neurons of heterozygous *PRKN* variant carriers. **a** Representative Western blot showing MFN2 and TOM70 protein levels in the hiPSC-derived neurons under basal conditions (−) and treated with 20 µM CCCP for 15 h (+). The asterisk indicates the monoubiquitinated form of MFN2. **b**, **c** Densitometric analysis of protein bands of a PD patient with homozygous *PRKN* mutations (*PRKN*++/PD+), the carriers group (*PRKN*+/PD−) comprising two heterozygous carriers (*PRKN*+/PD− 1 and *PRKN*+/PD− 2, respectively color coded) and the control group (representing the mean values of three control lines per experiment). MFN2 and TOM70 levels were analyzed with the indicated antibodies, and GAPDH was used as a loading control. Molecular mass markers are in kilodaltons (kDa). For both OMM proteins, levels were calculated as the ratio of MFN2 or TOM70 upon CCCP treatment over the levels of MFN2 or TOM70 under basal conditions (non-treated, NT). *n* = 3–4 independent experiments, respectively. The error bar represents the mean ± SEM. Significance levels were determined using one-way ANOVA and Tukey’s post hoc test to correct for multiple comparisons. *p ≤ 0.05, **p ≤ 0.01, ***p ≤ 0.001. ns not significant.

The general ubiquitination of outer mitochondrial membrane (OMM) proteins, such as Mitofusin-2 (MFN2), by Parkin is one of the early steps in mitophagy^30,44–46^, and Mitofusin-2 abundance was found to be decreased upon mitochondrial depolarization in induced neurons with endogenous Parkin levels^47^. We, therefore, evaluated MFN2 protein levels upon induction of mitochondrial depolarization with CCCP in hiPSC-derived neurons (Fig. 6a), which were revealed to be significantly higher in the homozygous *PRKN*-PD line and the heterozygous variant carrier lines, compared to the controls (90.2 ± 8.4 vs. 69.14 ± 8.4 vs. 41.9 ± 8.4; *p* = 0.016, *p* = 0.0007, respectively) (Fig. 6b), while there was no difference at basal conditions (data not shown). Moreover, it was possible to observe a band above the non-modified MFN2 in the controls and heterozygous variant carriers, possibly corresponding to the monoubiquitinated form of MFN2 (Fig. 6a). As an additional OMM protein, we further analyzed the levels of the mitochondrial import receptor subunit TOM70 (Fig. 6a), which upon depolarization revealed a trend towards increased levels for the heterozygous carrier group compared to the control group (p = 0.101; 59.55 ± 12 vs. 31.03 ± 12.2 respectively), and a significant increase for the homozygous *PRKN*-PD patient neurons (*p* = 0.002), as compared to the controls (100.2 ± 14.1 vs. 31.03 ± 12.2) (Fig. 6c). These results indicate that carrying a variant in *PRKN* leads to elevated outer membrane protein levels under stress conditions, thus possibly suggesting reduced ubiquitination, which might negatively impact mitophagy initiation or a general increase in mitochondrial mass.

### Mitochondrial DNA analyses in non-manifesting individuals carrying a pathogenic heterozygous variant in *PRKN*

We evaluated mitochondrial DNA (mtDNA) copy number in the blood of non-manifesting carriers (*n* = 341) of 17 pathogenic heterozygous *PRKN* variants, identified previously^11^, compared to controls (*n* = 8445, PD gene mutation-negative). This analysis suggests a trend towards higher levels in the group of variant carriers (*p* = 0.067, median carriers 142.2 vs. median of controls 134.95, respectively) (Supplementary Fig. 2a), while in the derived LCLs of a subgroup of 11 variant carriers and nine controls, no difference was detected (*p* = 0.602, median carriers 1970 vs. median controls 2104, respectively) (Supplementary Fig. 2d). The same was true for the mtDNA copy number of a subset of hiPSC-derived neurons of three controls, two variant carriers, and two *PRKN*-PD patients (5140 vs. 3309 vs. 3523, respectively) (Supplementary Fig. 2e). Comparison of blood samples of also a small group of 29 idiopathic PD (iPD) patients showed a trend for reduced mtDNA copy number levels (*p* = 0.053, median iPD 112.08 vs. median heterozygous carriers 142.2). Additionally, mtDNA-associated 7S DNA levels and mtDNA major arc deletion levels were assessed in a smaller group of heterozygous *PRKN* mutation carriers (*n* = 79) and controls (*n* = 77), where no differences were detected between groups (*p* = 0.53, 1.055 vs. 1.048, respectively and *p* = 0.73, 1.054 vs. 1.048, respectively) (Supplementary Fig. 2b, c).

## Discussion

In the present study, we show that non-manifesting individuals carrying a pathogenic heterozygous variant in *PRKN* present molecular phenotypes related to mitochondrial function. In peripheral blood of unaffected carriers of 17 heterozygous *PRKN* variants and controls, identified previously in the population-based CHRIS study^11^, a group comparison suggested a possible trend towards increased mtDNA copy number in heterozygous *PRKN* variant carriers. Several markers of mitochondrial functionality reveal significant differences between controls, non-manifesting carriers of a single pathogenic variant, and affected carriers of two variants in two cellular models derived from the investigated individuals. These mitochondrial alterations might, therefore, further support an increased vulnerability in heterozygous variant carriers and might be useful for identifying individuals at possibly greater risk of (subclinical) health problems or at worse, disease development.

Although PD is principally a neurodegenerative disorder, mounting data reveal molecular phenotypes in peripheral tissues suggesting the initiation of the pathology in the periphery. Of relevance, several studies show clear functional impairments in blood cells of iPD patients^48–51^. Furthermore, abnormalities in mitochondrial function have also been reported in peripheral tissues of biallelic *PRKN*-linked PD patients^52–55^. Our data collected in LCLs are characterized by enhanced mitochondrial respiration in non-manifesting individuals carrying a heterozygous *PRKN* exon 7 deletion in the routine (basal) respiratory state. Of note, a previous study reported a dramatically elevated basal mitochondrial respiration in iPD LCLs, which was observed independently of patient age, disease progression, and disease severity, suggesting an early and stable switch to a hyperactive state^56^. More recently, whole-cell transcriptomes and proteomes of iPD and healthy control LCLs showed downregulated transcripts for mitochondrial respiratory chain complexes but upregulated proteins, thereby suggesting that the elevated mitochondrial respiration observed in iPD LCLs is translationally regulated^57^. Interestingly, two studies performed in *PRKN*-mutant skin fibroblasts from PD patients also reported a higher mitochondrial respiratory rate compared to controls^58,59^. Our findings indicating an upregulation of basal mitochondrial respiration in LCLs of heterozygous *PRKN* variant carriers are suggestive of a cellular compensatory mechanism to overcome the changes occurring upon the presence of a *PRKN* variant, aiming to preserve mitochondrial function. Furthermore, these results may suggest mitochondrial hyperactivity in peripheral tissues as an early feature of mitochondrial dysfunction in PD. As expected, the heterozygous carrier group also presented an increased production of mROS, supporting the theory that elevated mROS may result from increased electron transport rates. Likewise, elevated ROS coupled with increased mitochondrial respiration was reported in iPD LCLs^56^. Conversely, a recent study conducted in *PRKN*-mutant LCLs reported an oxidative stress phenotype only upon treatment with toxins^60^. This suggests that a variant in *PRKN* may confer vulnerability to the system when subjected to environmental insults. In a new study^61^, an analysis of a larger set of *PRKN*/*PINK1* biallelic carrier PD patients and heterozygous *PRKN*/*PINK1* carriers both with and without PD symptoms, mtDNA heteroplasmy along with major arc deletions and 7S DNA were identified as potential disease manifestation markers for *PINK1*/*PRKN* PD. As mtDNA heteroplasmy is reliably measurable, and as the results support the direct correlation between overt phenotype and mtDNA alterations, performing blood-based deep mtDNA sequencing may be an important additional test to consider, especially in asymptomatic carriers. Increased mtDNA mutation load has functional consequences, which may provide additional functional assays alongside mtDNA heteroplasmy measurements. In the study sample reported here, while for mtDNA-associated 7S DNA levels and mtDNA major arc deletions, there was no difference between groups, the direction of the effect for both traits was in the same direction as that observed in this larger study sample^61^. Furthermore, we also detected a trend for increased levels of mtDNA copy number in non-manifesting carriers of heterozygous *PRKN* variants. This may suggest an accumulation of mtDNA molecules as a consequence of an impaired mitochondrial clearance process or a compensatory increase in mitochondrial biogenesis due to the presence of a single variant in *PRKN*.

In hiPSC-derived neurons of non-manifesting heterozygous *PRKN* exon 7 deletion carriers, which might be more susceptible to the genetic insult due to their predominant reliance on oxidative phosphorylation^38,40,62–64^, we found an overall decreased OCR, indicating a significant difference when cells were forced to perform at maximal capacity. This goes in parallel with reduced OCR observed in a homozygous *PRKN-*PD line, confirming previous reports on deficits in mitochondrial respiration and complex I activity in Parkin-deficient human neurons^65–67^. These findings suggest a respiratory impairment in neurons of heterozygous *PRKN* variant carriers, possibly indicating that a disease phenotype may be unmasked when stressors are added to the single genetic variant. Furthermore, heterozygous carriers, as well as a patient carrying two *PRKN* variants, exhibited increased levels of mitochondrial-derived oxidants, thus corroborating a mitochondrial phenotype in the absence or partial deficiency of Parkin. Increased oxidative stress levels were reported previously in Parkin-deficient iPSC-derived neurons^64,68–70^ and might give rise to a compensatory cellular response of antioxidant effectors such as catalase and SOD1/2^71^. Interestingly, our results suggest a reduction of mitochondrial SOD2 in complete or partial deficiency of Parkin. Accordingly, a decreased activity of SOD2 in fibroblasts of PD patients with *PRKN* variants, paired with increased mROS levels, was shown previously^72^. Furthermore, we observed a trend toward an increased mitochondrial network fragmentation in the neurons of the heterozygous variant carriers, and an increased fragmentation in the homozygous *PRKN*-PD line, which confirms previous data^65,66,70,73–75^. These data coincide with the altered mitochondrial function observed for the heterozygous *PRKN* carrier group as well as for the *PRKN*-PD line. The role of *PRKN* variants in mitophagy was studied broadly in cells overexpressing Parkin but was also confirmed in hiPSC-derived neurons with endogenous Parkin levels^64,65,69,73,76^. As expected, upon mitochondrial membrane depolarization, we found the levels of OMM proteins and Parkin substrates significantly increased in the neurons of the homozygous *PRKN*-PD line compared to the control neurons, consistent with impaired Parkin-dependent degradation, as observed previously for both *PRKN* and *PINK1* knockout animals and mutant human fibroblasts^45,46^. Remarkably, however, we were able to also observe an increase of OMM proteins in the hiPSC-derived neurons of non-manifesting individuals carrying a heterozygous *PRKN* variant, compatible with reduced Parkin activity. Interestingly, comparable results were obtained in fibroblasts of affected heterozygous *PRKN* variant carriers, excluding a second cryptic pathogenic variant^15^.

Altogether, when assessing mitochondrial function in the hiPSC-derived DA neuron model, we were able not only to confirm an impaired mitochondrial phenotype observed previously in hiPSC-derived DA neurons of homozygous *PRKN*-PD patients, but also to observe changes in mitochondrial homeostasis in clinically unaffected individuals carrying a heterozygous variant in *PRKN*, although these data need further confirmation since they are based on only two variant carriers. In addition, all measurements were performed on a mixed population of neurons, hence subtle differences in TH^+^ neurons may be lost. Based on the results in LCLs and hiPSC-derived neurons, we postulate that a beginning disease process could be characterized by hyperactive respiration in peripheral, rapidly turned over LCLs, where mitochondria seem to react to the insult, possibly as a compensatory mechanism, while in post-mitotic neurons, the damage of the insult might accumulate over time, contributing eventually to disease manifestation. An additional difference between the LCL and iPSC-derived neuronal models might be based on their preferential reliance upon the glycolysis and oxidative phosphorylation pathways for energy production. Although EBV strongly induces oxidative phosphorylation upon B-cell infection and transformation into LCLs^77^, these cells might rely more on glycolysis^78^, and defects in the glycolysis pathway, providing pyruvate for the tricarboxylic acid cycle, might force a shift from glycolysis to mitochondrial respiration in the heterozygous *PRKN* variant carrier group. Overall, we consider this study as a proof of concept for detecting molecular phenotypes in cellular models derived from heterozygous *PRKN* variant carriers. In additional studies, it will be interesting to correlate molecular phenotypes with subclinical (prodromal) phenotypes. For this, larger sample sizes will be needed^79,80^.

In summary, two different cellular models of non-manifesting individuals carrying a heterozygous exon 7 deletion in *PRKN* present detectable mitochondrial defects, albeit with small effects. Notably, the altered molecular phenotype in the unaffected heterozygous variant carriers is seemingly milder as compared to PD patients lacking Parkin, possibly suggesting a gene dosage effect. This likely mirrors the clear dosage effect observed in mtDNA heteroplasmy, major arc deletions, and 7S DNA reported recently^61^. However, questions as to what extent these alterations can affect the overall function of the mitochondria to the point of translating into disease remain to be investigated. Additional factors, including age and environment, may push single variant carriers over a gradual threshold, whereby carriers might convert from presenting mild, barely observable subclinical phenotypes to becoming symptomatic. The gradient of mitochondrial function markers detected between heterozygous and homozygous *PRKN* variant carriers suggests that there may indeed be a threshold of disrupted mitochondrial function, past which a *PRKN* variant carrier may convert to a disease state. It will therefore be important to study the degree to which mitochondrial protection factors may represent a strategy to rescue mitochondrial function in the cell models of heterozygous variant carriers^81,82^. Such a strategy may delay, or possibly prevent, the conversion to clinical phenotype.

In conclusion, our findings show an altered molecular phenotype in non-manifesting carriers of a pathogenic, heterozygous *PRKN* variant, characterized by disparities at the level of mitochondrial function in two different cellular models derived from the variant carriers. To adequately understand the individual risk for disease conversion, there is a need for more longitudinal studies with putatively healthy individuals carrying single *PRKN* variants, more in-depth clinical phenotyping, and measurements of the molecular markers described in this study.

## Methods

### Ethics statement

The CHRIS study is a joint initiative with the South Tyrolean healthcare system conducted under the leadership of the Institute for Biomedicine, Eurac Research, and was approved by the Ethics Committee of the Healthcare System of the Autonomous Province of Bozen/Bolzano (Prot. 0043339-BZ, 28/04/2011). The generation of iPSCs was approved by the Ethics Committee of the Healthcare System of the Autonomous Province of Bozen/Bolzano (Prot. 0042955-BZ, 06/04/2018). Study participants provided their written informed consent to participate in this study.

### Study participants

Study participants were enrolled in the Cooperative Health Research in South Tyrol (CHRIS) study^83^. mtDNA copy number was quantified in 341 non-manifesting carriers of 17 pathogenic heterozygous *PRKN* variants (*PRKN*+/PD−), identified previously^11^, 29 idiopathic PD (iPD) patients, and 8445 controls. Analyses for mtDNA major arc deletions and mtDNA transcription initiation were conducted in a subset of 79 heterozygous *PRKN* variant carriers and 77 controls. Furthermore, mitochondrial performance was assessed in cellular models derived from 21 CHRIS study participants originating from four families, who were recalled and deep-phenotyped based on their genetic profile^18^. For these 21 individuals, lymphoblastoid cell lines (LCLs) and/or human induced pluripotent stem cell (hiPSC)-derived neurons were generated (Supplementary Table 1). From these, 11 carried a heterozygous *PRKN* variant (exon 7 deletion, *PRKN*+/PD−), which is among the most frequent mutations identified, and ten were control individuals from the same families (matched by close age), without *PRKN* variants. Family 1 included six variant carriers and five controls, family 2 one carrier and one control, and families 3 and 4 included two carriers and two controls each. None of the heterozygous *PRKN* mutation carriers fulfilled the diagnostic criteria for PD, which was assessed with a screening questionnaire for parkinsonism^84^ and by clinical examination of the individuals, for which cell lines were established^18^. In the neurological assessment, no difference between groups was observed for motor or non-motor symptoms and *substantia nigra* hyperechogenicity, but sensor-based quantification of movements under specific task challenge allowed discrimination of unaffected heterozygous mutation carriers from mutation-free controls (larger sample set including the 21 individuals with cell lines generated in this study)^18^. hiPSCs were generated for two heterozygous *PRKN* mutation carriers and two controls, all from the same family^41^; both mutation carriers (*PRKN*+/PD− 1, *PRKN*+/PD− 2) presented with *substantia nigra* hyperechogenicity and subtle motor signs, one control (Control 2) did not show any subclinical or prodromal signs, and the other control (Control 3) was positive for *substantia nigra* hyperechogenicity. Additionally, hiPSC lines from a *PRKN*-PD patient with a homozygous mutation (*PRKN*++/PD+)^66,85^ and a control individual (https://cells.ebisc.org/STBCi033-B/) were included in the functional analyses for comparison. Demographic and basic clinical data for individuals with cell lines are summarized in Supplementary Table 1. An overview of all individuals included in the study is depicted in Fig. 1.

### Generation of lymphoblastoid cell lines and culture

Peripheral blood was collected in EDTA-buffered collection tubes, and peripheral blood mononuclear cells (PBMCs) were isolated using Histopaque—1077 cell separation medium (Sigma Aldrich) and Leucosep tubes (Greiner Bio-one), according to manufacturer’s instructions. Lymphoblastoid cell lines (LCLs) were established by infecting B-cells from the PBMCs with Epstein Barr Virus (EBV). Briefly, PBMCs were incubated with 2 mL of culture supernatant from B95.8 cells (marmoset cell line) expressing EBV, at 37 °C in a 5% CO_2_ atmosphere for 2.5 h. Then, cells were kept in RPMI 1640 (Roswell Park Memorial Institute) culture medium (Biowest), supplemented with 10% Fetal bovine serum (FBS) (Sigma Aldrich) and 1% penicillin-streptomycin (Thermo Fisher Scientific), containing phytohemagglutinin (PHA) (Sigma Aldrich) to induce the secretion of T-cell-assisted B-cell growth factors and the removal of T-cells^86,87^. One week after infection, clusters were visible, and PHA was withdrawn. For experiments, LCLs were seeded at a concentration of 3 × 10^5^ cells/mL and passaged at intervals of three days. Cells were kept in culture in a saturated, humidified atmosphere at 37 °C and 5% CO_2_ for a maximum of 10 passages.

### Flow cytometry

Flow cytometry was used to assess mitochondrial membrane potential (MMP) and the production of mitochondrial reactive oxygen species (mROS) in LCLs. The potentiometric dye tetramethylrhodamine methyl ester (TMRM Image-iT™; Thermo Fisher Scientific) was used as an MMP indicator with a final concentration of 100 nM. To exclude dead cells from the analyzed samples, VivaFix™ Cell Viability Assay (Biorad) was used together with TMRM. Labeled cells were counted based on TMRM intensity, which corresponds to the levels of their MMP. As a positive control, to represent the background activity of TMRM staining, cells were treated with CCCP (50 µM, 1 h), which completely dissipates the MMP. To assess mROS production (mitochondrial superoxide), MitoSOX™ Red reagent (Thermo Fisher Scientific) was used. Cells were co-stained with 2.5 µM MitoSox Red and 200 nM Mito Tracker™ Green (MTG, Thermo Fisher Scientific) to select the cell population of interest for the acquisition and analysis. Stained LCLs were interrogated using an S3e Cell Sorter (Biorad), operated by the ProSort™ Software, version 1.6. The fluorescence intensity of 20,000 events was quantified and analyzed using the FlowJo™ v10.6.1 software. Fluorescence intensity was determined based on the gating strategy described in the supplementary information (Supplementary Fig. 3).

### High-resolution respirometry in LCLs

Mitochondrial oxygen consumption was measured in LCLs by means of high-resolution respirometry (HRR) with the Oxygraph-2K (O2K, Oroboros Instruments, Innsbruck, Austria). Experiments were carried out under normoxic conditions (O_2_ concentration at and below air saturation). Air calibration was performed according to the manufacturer´s instructions, and the temperature was set at 37 ± 0.001 °C (electronic Peltier regulation) under constant stirring at 750 rpm. Data recording was performed in real-time using the DatLab7 software (Oroboros Instruments), which was also used for post-experimental analysis. Experiments were performed in MiR05 respiration medium (Oroboros Instruments), and manual titration of mitochondrial inhibitors and uncouplers was performed using Hamilton syringes (customized by Hamilton®). Briefly, 5 ×10^6^ LCLs were harvested and added to each O2K chamber. Mitochondrial respiration activity of intact LCLs was characterized by using the following multiple substrate-uncoupler-inhibitor titration protocol (SUIT-003, BioBlast): (1) ROUTINE corresponds to physiological respiration (depending on endogenous substrates), (2) LEAK respiration is induced by adding the ATP synthase inhibitor oligomycin (Omy, 10 nM; Sigma Aldrich) and corresponds to the non-phosphorylating resting state, when oxygen flux is compensating for the proton leak, (3) maximal capacity of the electron transfer system determined after uncoupling by stepwise titration (usually 1–2 steps) of the protonophore carbonyl cyanide-p-trifluoromethoxyphenylhydrazone (FCCP, U, 0.5 μM each step; Sigma Aldrich), (4) residual oxygen consumption achieved after the injection of complex I inhibitor rotenone (Rot, 0.5 μM; Sigma Aldrich), (5) addition of succinate (S, 10 mM; Acros Organics) to test the intactness of the cell membrane, and (6) residual oxygen consumption after the injection of the complex III-inhibitor antimycin A (Ama, 2.5 μM; Sigma Aldrich). Respiratory fluxes were corrected for instrumental background considering the oxygen consumption by the sensors and the oxygen diffusion of the chamber. Respiration related to ATP turnover was calculated as the difference between ROUTINE and LEAK respiration (R-L), and spare respiratory capacity was determined as the difference between maximal respiration and routine respiration (ET-R). Absolute respiration values were normalized for the total number of cells per chamber and citrate synthase activity.

### Citrate synthase (CS) activity

CS was assayed spectrophotometrically at 412 nm and 30 °C on the EnVision 2105 Multimode Plate Reader (Perkin-Elmer), according to a published protocol^88^ with slight modifications, where the final concentrations for the reaction were acetyl-CoA 100 µM, oxalacetate 100 µM and 5,5′-dithiobis- (2-dinitrobenzoic acid) 100 µM in tris chloride buffer, pH 8 (100 mM). Following respirometry in the O2K, LCLs were removed from the chamber, and an aliquot was snap-frozen. CS activity measurements were performed in duplicates for each respirometry experiment, and averaged values were used for the normalization of respirometry results.

### Differentiation of hiPSCs into dopaminergic neurons

The direct differentiation of hiPSCs into dopaminergic (DA) neurons was conducted as described previously^89,90^, with minor modifications^66^. Human iPSC colonies were disaggregated into single cells using accutase and replated onto matrigel (BD)-coated dishes in mTeSR™ 1 complete medium, supplemented with 10 μM ROCK inhibitor Y-27632 (Miltenyi Biotech) at a density of 57,000–62,000 cells/cm^2^. Differentiation was started once hiPSCs reached a confluence of 90% by adding knockout serum replacement (KSR) medium supplemented with SMAD pathway inhibitors SB431542 (SB, Miltenyi Biotech) and LDN-193189 (LDN, StemMACS). On days 1 to 5, KSR medium was added to the cells in the presence of SB, LDN, recombinant Human Sonic Hedgehog (SHH, R&D System), recombinant Human FGF-8a (FGF8, R&D System), and Purmorphamine (Pu, StemMACS). The Wnt pathway activator molecule CHIR99021 (CH, StemMACS) was included from days 3–12. During days 6–10 of differentiation, increasing amounts of Neurobasal medium plus B27 supplement (NB-B27 medium, Thermo Fisher Scientific) was added to the KSR medium (25, 50, and 75%), and upon day 7, SHH, FGF8, and Pu were withdrawn. On day 11, maturation of DA neurons was initiated by adding recombinant Human BDNF (Peprotech), ascorbic acid (Sigma Aldrich), recombinant Human TGF-ß3 (Peprotech), cyclic-AMP (EnzoLifescience) and DAPT (Tocris). Cells were passaged between days 12 and 15 *en bloc*^90^, and between days 20 and 25 of differentiation, they were replated as single cells onto plastic dishes or live imaging chamber slides, previously coated with poly-D-lysine (Sigma Aldrich) and laminin (Sigma Aldrich), for western blot and imaging experiments. For oxygen consumption rate (OCR) assay experiments, neurons were replated en bloc between days 12 and 15 and replated at day 30 on the 96-well plates for the OCR assay, where they were stabilized and further differentiated until day 34. By using this differentiation protocol, a yield of ~40% of the DA neuron marker tyrosine hydroxylase (TH)-expressing neurons is obtained, as shown previously^66^. Detailed experimental procedures are available in the supplementary material.

### Mitochondrial oxygen consumption rate in hiPSC-neurons

Mitochondrial oxygen consumption rate (OCR) was measured using the Extracellular O_2_ Consumption Assay Kit (Abcam, ab197243) according to the manufacturer’s instructions. At day 31 of differentiation, 2 × 10^5^ hiPSC-derived neurons were seeded on a 96-well cell culture plate, previously coated with poly-d-lysine (Sigma Aldrich) and laminin (Sigma Aldrich) and incubated in a CO_2_ incubator at 37 °C with NB-B27 medium supplemented with neuronal differentiation factors. Cells recovered for 72 h prior to performing the OCR measurements. For the assay, the medium was replaced with 150 µl of fresh NB-B27 medium for routine (basal) OCR measurements, or with 150 µl of NB-B27 medium containing 2.5 µM FCCP for maximal respiration measurements. Next, 10 μL of Extracellular O_2_ consumption reagent were added to each well, except to the blank control, and 100 µl of high-sensitivity mineral oil (pre-heated at 37 °C) were added to limit back diffusion of ambient oxygen. Fluorescence intensities were measured using the EnVision 2105 Multimode Plate Reader (Perkin-Elmer), pre-heated at 37 °C, in 2-min- intervals for a total of 200 min, at excitation/emission wavelengths = 355/642 nm. Respiration of cells results in oxygen depletion from the surrounding environment, causing an increase of fluorescence signal. Immediately after performing the Extracellular O_2_ Consumption Assay, the CyQuant™ proliferation assay (Thermo Fisher Scientific) was employed to determine the number of live cells in each well, according to the manufacturer´s instructions. Fluorescence intensity was corrected with the blank control, and OCR was determined by selecting the linear portion of the signal profile (avoiding any initial lag or subsequent plateau) and applying the linear regression to determine the slope. The OCR calculated for each well was normalized to the cell number determined by CyQuant fluorescence dye. Fluorescence intensities detecting OCR are expressed as relative fluorescence units (RFU) versus time (min).

### Live Imaging

To assess MMP, mROS production, and mitochondrial morphology in hiPSC-derived neurons under live imaging conditions, 2 × 10^5^ cells per well were seeded as single cells on an eight-well live imaging chamber slide (ibidi GmBH) previously coated with poly-d-lysine (Sigma Aldrich) and laminin (Sigma Aldrich). Neurons were imaged at day 40 of differentiation. Cells were incubated with a staining solution containing either 100 nM TMRM to assess MMP, 2.5 µM MitoSox Red to evaluate ROS production, or 100 nM MTG to evaluate mitochondrial morphology, at 37 °C and 5% CO_2_ for 30 min. As a positive control, to represent the background activity of TMRM staining with the concentration used, cells were treated with CCCP (10 µM, 45 min), which completely dissipates the MMP. After washing with pre-warmed HBSS 1X, cells were directly imaged in HBSS at 37 °C and 5% CO_2_ using a Leica SP8-X confocal microscope (Leica Microsystems, LAS-X acquisition software), with hybrid detectors and a 63X oil immersion objective. For mean intensity measurements of TMRM and MitoSox Red in thresholded images, Image J software was used (https://imagej.nih.gov/ij/index.html). Mitochondrial fragmentation, assessed based on MTG staining, was calculated using the filament function in Imaris software (version 9.6.0, Bitplane) (Oxford Instruments): for each field, the total length of the modeled mitochondrial network was calculated and divided by the number of unconnected parts.

### Immunofluorescence

For immunocytochemical analysis, differentiated neurons were grown on a 16-well format chamber slide System Nunc™ Lab-Tek™ (Thermo Fisher Scientific), previously coated with poly-d-lysine (Sigma Aldrich) and laminin (Sigma Aldrich), and fixed in a 4% paraformaldehyde solution. Next, cells were permeabilized in 0.5% Triton X-100—PBS and blocked with a blocking solution (3% BSA in PBS) at RT. Immunostaining was performed by the addition of primary antibodies to co-stain the DA neuronal marker TH (rabbit anti-TH, Merck Millipore MAB318) and the class III member of the beta-tubulin family (mouse anti-TUJ1, Biolegend). Subsequently, cells were incubated with the appropriate fluorescently labeled secondary antibodies (goat anti-mouse Alexa Fluor 488-conjugated, goat anti-rabbit Alexa Fluor 555-conjugated, Thermo Fisher Scientific), nuclei were stained by adding NucBlue™ Fixed Cell Stain (Thermo Fisher Scientific), and the slides were mounted with Dako Fluorescence mounting medium. Images were acquired using a Leica SP8-X confocal microscope (Leica Microsystems, LAS-X acquisition software), with hybrid detectors and a 63X oil immersion objective.

### Treatment conditions

In order to assess protein levels of MFN2 (Mitofusin-2) and TOM70 (mitochondrial import receptor subunit) in hiPSC-derived neurons upon dissipation of the mitochondrial membrane potential, treatment with 20 µM carbonyl cyanide 4-(trifluoromethoxy) phenylhydrazone (CCCP) (Sigma Aldrich) was performed for 15 h.

### Western blot analysis

For Western blot analysis, whole-cell homogenates were used. Harvested cells were resuspended in cold RIPA buffer (Thermo Fisher Scientific) supplemented with protease inhibitors cOmplete™ Protease Inhibitor Cocktail (Roche) and phosphatase inhibitors PhosSTOP EASYpack phosphatase inhibitors (Roche). Protein concentrations were assessed using a BCA Protein Assay Kit (Thermo Fisher Scientific), and 10 µg of total protein lysates were loaded per well on a NuPAGE 4–12% Bis-Tris SDS-PAGE gel (Thermo Fisher Scientific). After electrophoresis, proteins were transferred onto a PVDF membrane (BioRad) and probed with antibodies raised against MFN2 (Abcam, ab56889), TOM70 (Abcam, ab135602), SOD2 (Cell Signaling, 13141 S), Parkin (Abcam, 77924), and GAPDH (Millipore, MAB374). Subsequently, the blots were incubated with the corresponding secondary antibodies, and target proteins were detected by enhanced chemiluminescence using Clarity™ ECL Western Kit (BioRad). The chemiluminescence signal was detected using the ChemiDoc™ Touch Imaging System (BioRad) and quantified by densitometry using Image Lab 6.0 analyzer software (BioRad). Optical density values assessed for target proteins were normalized by the indicated loading control. For each figure, all blots were processed in parallel and originated from the same experiment.

### Mitochondrial DNA analyses

mtDNA was extracted from whole blood and cultured cells (LCLs and hiPSC-derived neurons) using a standard procedure for extraction of genomic DNA. mtDNA copy number was measured using a duplex quantitative real-time PCR assay to simultaneously detect the mtDNA gene *tRNA-Leu* and the single-copy nuclear gene *beta-2-microglobulin* (*B2M*), as previously described in ref. ^91^. mtDNA copy number data of the CHRIS study participants were available from a previous study^91^. mtDNA transcription-associated 7S DNA levels and major arc deletions were assessed using a real-time PCR assay based on TaqMan chemistry^34^. Probes were used for the quantification of mtDNA sequences in the minor arc gene *NADH-dehydrogenase 1* (*MT-ND1*), the major arc gene *NADH dehydrogenase 4* (*MT-ND4*), and the D-loop, located within the non-coding region (NCR) of mtDNA. A region of mtDNA-7S was amplified using the forward primer 5′-CCCACACGTTCCCCTTAAATAA-3′ and the reverse primer 5′-CGTGAGTGGTTAATAGGGTGATAGAC-3′; a region of *MT-ND1* was amplified using the forward primer 5′-CCCTAAAACCCGCCACATCTAC-3′ and the reverse primer 5′-GAGCGATGGTGAGAGCTAAGGT-3′; and a region of *MT-ND4* was amplified using the forward primer 5′-CCATTCTCCTCCTATCCCTCAAC-3′ and the reverse primer 5′-CACAATCTGATGTTTTGGTTAAACTATATTT-3′. Probe sequences were: ALEXA594-5′-ACATCACGATGGATCAC-3′ -MGB for D-Loop, VIC-5′ -CCATCACCCTCTACATCACCGCCC-3′ -QSY for *MT-ND1* and FAM-5′ -CCGACATCATTACCGGGTTTTCCTCTTG-3′ -QSY for *MT-ND4*. The real-time PCR was performed using the following conditions: 95 °C for 10 s, 45 cycles of 95 °C for 15 s, 60 °C for 30 s, and 72 °C for 3 sec.

### Statistical analyses

Statistical analyses were performed using R version 4.1.2 and GraphPad Prism 9. One-way ANOVA was used in experiments comparing three groups, followed by Tukey’s post hoc test to correct for multiple comparisons. For analyzing differences between the two experimental groups, the non-parametric Mann–Whitney *U*-test was utilized. The threshold for significance was set at *p* < 0.05. All experiments were performed in a minimum of three independent biological replicates.

### Reporting summary

Further information on research design is available in the Nature Research Reporting Summary linked to this article.

## Supplementary information

Supplementary Information

Reporting Summary

## Data Availability

All data generated during this study are included in this article and its supplementary information files. Genotype data are not readily available because the data are part of a large population-based study (CHRIS study), and the informed consent provided by the study participants does not allow the upload of individual-level genetic data to public repositories.

## References

[1] Bloem, B. R., Okun, M. S. & Klein, C. Parkinson’s disease. *Lancet*. 10.1016/S0140-6736(21)00218-X (2021).

[2] Kouli, A., Torsney, K. M. & Kuan, W. L. In *Parkinson’s Disease: Pathogenesis and Clinical Aspects* (eds Stoker, T. B. & Greenland, J. C.) Ch. 1, (Codon Publications, 2018). 30702835

[3] Forno LS (1996). Neuropathology of Parkinson’s disease. J. Neuropathol. Exp. Neurol..

[4] Moustafa AA (2016). Motor symptoms in Parkinson’s disease: a unified framework. Neurosci. Biobehav. Rev..

[5] Blauwendraat C, Nalls MA, Singleton AB (2020). The genetic architecture of Parkinson’s disease. Lancet Neurol..

[6] Park JS, Davis RL, Sue CM (2018). Mitochondrial dysfunction in Parkinson’s disease: new mechanistic isights and therapeutic perspectives. Curr. Neurol. Neurosci. Rep..

[7] Domingo A, Klein C (2018). Genetics of Parkinson disease. Handb. Clin. Neurol..

[8] Kasten M (2018). Genotype-phenotype relations for the Parkinson’s disease genes Parkin, PINK1, DJ1: MDSGene systematic review. Mov. Disord..

[9] Lesage, S. et al. Characterization of recessive Parkinson’s disease in a large multicenter study. *Ann. Neurol*. 10.1002/ana.25787 (2020). 10.1002/ana.25787 PMC8944279 33045815

[10] Pramstaller PP (2005). Lewy body Parkinson’s disease in a large pedigree with 77 Parkin mutation carriers. Ann. Neurol..

[11] Castelo Rueda MP (2021). Frequency of heterozygous Parkin (PRKN) variants and penetrance of Parkinson’s disease risk markers in the population-based CHRIS cohort. Front. Neurol..

[12] Klein C, Lohmann-Hedrich K, Rogaeva E, Schlossmacher MG, Lang AE (2007). Deciphering the role of heterozygous mutations in genes associated with parkinsonism. Lancet Neurol.

[13] Huttenlocher J (2015). Heterozygote carriers for CNVs in PARK2 are at increased risk of Parkinson’s disease. Hum. Mol. Genet..

[14] Lubbe SJ (2021). Assessing the relationship between monoallelic PRKN mutations and Parkinson’s risk. Hum. Mol. Genet..

[15] Zhu, W. et al. Heterozygous PRKN mutations are common but do not increase the risk of Parkinson’s disease. *Brain*. 10.1093/brain/awab456 (2022). 10.1093/brain/awab456 PMC9423714 35640906

[16] Yu E (2021). Analysis of heterozygous PRKN variants and copy-number variations in Parkinson’s disease. Mov. Disord..

[17] Weissbach A (2017). Influence of L-dopa on subtle motor signs in heterozygous Parkin- and PINK1 mutation carriers. Parkinsonism Relat. Disord..

[18] Prasuhn J (2021). Task matters - challenging the motor system allows distinguishing unaffected Parkin mutation carriers from mutation-free controls. Parkinsonism Relat. Disord..

[19] Binkofski F (2007). Morphometric fingerprint of asymptomatic Parkin and PINK1 mutation carriers in the basal ganglia. Neurology.

[20] Hilker R (2001). Positron emission tomographic analysis of the nigrostriatal dopaminergic system in familial parkinsonism associated with mutations in the parkin gene. Ann. Neurol..

[21] Guo JF (2011). Clinical features and [11C]-CFT PET analysis of PARK2, PARK6, PARK7-linked autosomal recessive early onset Parkinsonism. Neurol. Sci..

[22] Pavese N (2009). Nigrostriatal dysfunction in homozygous and heterozygous parkin gene carriers: an 18F-dopa PET progression study. Mov. Disord..

[23] Santos M, Morais S, Pereira C, Sequeiros J, Alonso I (2019). Parkin truncating variants result in a loss-of-function phenotype. Sci. Rep..

[24] Mouton-Liger F, Jacoupy M, Corvol JC, Corti O (2017). PINK1/Parkin-dependent mitochondrial surveillance: from pleiotropy to Parkinson’s disease. Front. Mol. Neurosci..

[25] Ge P, Dawson VL, Dawson TM (2020). PINK1 and Parkin mitochondrial quality control: a source of regional vulnerability in Parkinson’s disease. Mol. Neurodegener..

[26] Shimura H (2000). Familial Parkinson disease gene product, parkin, is a ubiquitin-protein ligase. Nat. Genet..

[27] Youle RJ, Narendra DP (2011). Mechanisms of mitophagy. Nat. Rev. Mol. Cell Biol..

[28] Trempe JF (2013). Structure of parkin reveals mechanisms for ubiquitin ligase activation. Science.

[29] Trinh D, Israwi AR, Arathoon LR, Gleave JA, Nash JE (2021). The multi-faceted role of mitochondria in the pathology of Parkinson’s disease. J. Neurochem..

[30] Gegg ME (2010). Mitofusin 1 and mitofusin 2 are ubiquitinated in a PINK1/parkin-dependent manner upon induction of mitophagy. Hum. Mol. Genet..

[31] Narendra D, Tanaka A, Suen DF, Youle RJ (2008). Parkin is recruited selectively to impaired mitochondria and promotes their autophagy. J. Cell Biol..

[32] Vives-Bauza C (2010). PINK1-dependent recruitment of Parkin to mitochondria in mitophagy. Proc. Natl Acad. Sci. USA.

[33] Shin JH (2011). PARIS (ZNF746) repression of PGC-1alpha contributes to neurodegeneration in Parkinson’s disease. Cell.

[34] Wasner, K. et al. Parkin deficiency impairs mitochondrial DNA dynamics and propagates inflammation. *Mov. Disord.*10.1002/mds.29025 (2022). 10.1002/mds.29025 35460111

[35] Pickrell AM (2015). Endogenous Parkin preserves dopaminergic substantia nigral neurons following mitochondrial DNA mutagenic stress. Neuron.

[36] Bruggemann N (2009). Frequency of heterozygous Parkin mutations in healthy subjects: need for careful prospective follow-up examination of mutation carriers. Parkinsonism Relat. Disord..

[37] Qadri R (2018). Alterations in mitochondrial membrane potential in peripheral blood mononuclear cells in Parkinson’s disease: potential for a novel biomarker. Restor. Neurol. Neurosci.

[38] Agostini M (2016). Metabolic reprogramming during neuronal differentiation. Cell Death Differ.

[39] Prigione A, Fauler B, Lurz R, Lehrach H, Adjaye J (2010). The senescence-related mitochondrial/oxidative stress pathway is repressed in human induced pluripotent stem cells. Stem Cells.

[40] Maffezzini C, Calvo-Garrido J, Wredenberg A, Freyer C (2020). Metabolic regulation of neurodifferentiation in the adult brain. Cell Mol. Life Sci..

[41] Castelo Rueda MP (2022). Generation and characterization of induced pluripotent stem cell (iPSC) lines of two asymptomatic individuals carrying a heterozygous exon 7 deletion in Parkin (PRKN) and two non-carriers from the same family. Stem Cell Res..

[42] Brand MD (2004). Mitochondrial superoxide: production, biological effects, and activation of uncoupling proteins. Free Radic. Biol. Med..

[43] Dias V, Junn E, Mouradian MM (2013). The role of oxidative stress in Parkinson’s disease. J. Parkinsons Dis..

[44] Tanaka A (2010). Proteasome and p97 mediate mitophagy and degradation of mitofusins induced by Parkin. J. Cell Biol..

[45] Rakovic A (2011). Mutations in PINK1 and Parkin impair ubiquitination of Mitofusins in human fibroblasts. PLoS ONE.

[46] Ziviani E, Tao RN, Whitworth AJ (2010). Drosophila parkin requires PINK1 for mitochondrial translocation and ubiquitinates mitofusin. Proc. Natl Acad. Sci. USA.

[47] Ordureau A (2020). Global landscape and dynamics of Parkin and USP30-dependent ubiquitylomes in iNeurons during mitophagic signaling. Mol. Cell.

[48] Nissen SK (2019). Alterations in blood monocyte functions in Parkinson’s disease. Mov. Disord..

[49] Scherzer CR (2007). Molecular markers of early Parkinson’s disease based on gene expression in blood. Proc. Natl Acad. Sci. USA.

[50] Smith AM (2018). Mitochondrial dysfunction and increased glycolysis in prodromal and early Parkinson’s blood cells. Mov. Disord..

[51] Vida C (2019). Lymphoproliferation impairment and oxidative stress in blood cells from early Parkinson’s disease patients. Int. J. Mol. Sci..

[52] Grunewald A (2010). Mutant Parkin impairs mitochondrial function and morphology in human fibroblasts. PLoS ONE.

[53] Mortiboys H (2008). Mitochondrial function and morphology are impaired in parkin-mutant fibroblasts. Ann. Neurol..

[54] Zilocchi M (2020). Exploring the impact of PARK2 mutations on the total and mitochondrial proteome of human skin fibroblasts. Front. Cell Dev. Biol..

[55] Muftuoglu M (2004). Mitochondrial complex I and IV activities in leukocytes from patients with parkin mutations. Mov. Disord..

[56] Annesley SJ (2016). Immortalized Parkinson’s disease lymphocytes have enhanced mitochondrial respiratory activity. Dis. Model. Mech..

[57] Annesley SJ, Allan CY, Sanislav O, Evans A, Fisher PR (2022). Dysregulated Gene Expression in Lymphoblasts from Parkinson’s Disease. Proteomes.

[58] Haylett W (2016). Altered mitochondrial respiration and other features of mitochondrial function in Parkin-mutant fibroblasts from Parkinson’s disease patients. Parkinsons Dis..

[59] Gonzalez-Casacuberta I (2019). Mitochondrial and autophagic alterations in skin fibroblasts from Parkinson disease patients with Parkin mutations. Aging.

[60] Ming F (2020). The PARK2 mutation associated with Parkinson’s disease enhances the vulnerability of peripheral blood lymphocytes to paraquat. Biomed. Res. Int..

[61] Trinh, J. et al. Mitochondrial DNA heteroplasmy distinguishes disease manifestation in PINK1/PRKN-linked Parkinson’s disease. *Brain* awac464 (2022). 10.1093/brain/awac464 PMC10316771 36478228

[62] Zheng X (2016). Metabolic reprogramming during neuronal differentiation from aerobic glycolysis to neuronal oxidative phosphorylation. Elife.

[63] O’Brien LC, Keeney PM, Bennett JP (2015). Differentiation of human neural stem cells into motor neurons stimulates mitochondrial biogenesis and decreases glycolytic flux. Stem Cells Dev..

[64] Schwartzentruber A (2020). Oxidative switch drives mitophagy defects in dopaminergic parkin mutant patient neurons. Sci. Rep..

[65] Kumar M (2020). Defects in mitochondrial biogenesis drive mitochondrial alterations in PARKIN-deficient human dopamine neurons. Stem Cell Rep..

[66] Zanon A (2017). SLP-2 interacts with Parkin in mitochondria and prevents mitochondrial dysfunction in Parkin-deficient human iPSC-derived neurons and Drosophila. Hum. Mol. Genet..

[67] Bogetofte H (2019). PARK2 mutation causes metabolic disturbances and impaired survival of human iPSC-derived neurons. Front. Cell Neurosci..

[68] Okarmus J (2021). Identification of bioactive metabolites in human iPSC-derived dopaminergic neurons with PARK2 mutation: Altered mitochondrial and energy metabolism. Stem Cell Rep..

[69] Yamaguchi A (2020). Identifying therapeutic agents for amelioration of mitochondrial clearance disorder in neurons of familial Parkinson disease. Stem Cell Rep..

[70] Chung SY (2016). Parkin and PINK1 patient iPSC-derived midbrain dopamine neurons exhibit mitochondrial dysfunction and alpha-synuclein accumulation. Stem Cell Rep..

[71] Filograna R, Beltramini M, Bubacco L, Bisaglia M (2016). Anti-oxidants in Parkinson’s disease therapy: a critical point of view. Curr. Neuropharmacol..

[72] Pacelli C (2011). Mitochondrial defect and PGC-1alpha dysfunction in parkin-associated familial Parkinson’s disease. Biochim. Biophys. Acta.

[73] Imaizumi Y (2012). Mitochondrial dysfunction associated with increased oxidative stress and alpha-synuclein accumulation in PARK2 iPSC-derived neurons and postmortem brain tissue. Mol. Brain.

[74] Shaltouki A (2015). Mitochondrial alterations by PARKIN in dopaminergic neurons using PARK2 patient-specific and PARK2 knockout isogenic iPSC lines. Stem Cell Rep..

[75] Yokota M (2021). Establishment of an in vitro model for analyzing mitochondrial ultrastructure in PRKN-mutated patient iPSC-derived dopaminergic neurons. Mol. Brain.

[76] Suzuki S (2017). Efficient induction of dopaminergic neuron differentiation from induced pluripotent stem cells reveals impaired mitophagy in PARK2 neurons. Biochem. Biophys. Res. Commun..

[77] Burton EM, Gewurz BE (2022). Epstein-Barr virus oncoprotein-driven B cell metabolism remodeling. PLoS Pathog..

[78] Darekar S (2012). Epstein-Barr virus immortalization of human B-cells leads to stabilization of hypoxia-induced factor 1 alpha, congruent with the Warburg effect. PLoS ONE.

[79] Fraenkel, J. R., Hyun, H.H., & Wallen, N.E. *How to Design and Evaluate Research in Education* 8th edn (McGraw Hill, 2012).

[80] Gall, M. D., Gall, J. P., & Borg, W. R. *Educational Research: An Introduction* 7th edn (Pearson Education Inc., 2003).

[81] Prasuhn J (2019). An omics-based strategy using coenzyme Q10 in patients with Parkinson’s disease: concept evaluation in a double-blind randomized placebo-controlled parallel group trial. Neurol. Res. Pract..

[82] Prasuhn J (2020). The use of vitamin K2 in patients with Parkinson’s disease and mitochondrial dysfunction (PD-K2): a theranostic pilot study in a placebo-controlled parallel group design. Front. Neurol..

[83] Pattaro C (2015). The Cooperative Health Research in South Tyrol (CHRIS) study: rationale, objectives, and preliminary results. J. Transl. Med..

[84] Pramstaller PP, Falk M, Schoenhuber R, Poewe W (1999). Validation of a mail questionnaire for parkinsonism in two languages (German and Italian). J. Neurol..

[85] Zanon A (2019). Generation of an induced pluripotent stem cell line (EURACi005-A) from a Parkinson’s disease patient carrying a homozygous exon 3 deletion in the PRKNgene. Stem Cell Res..

[86] Penno MB, Pedrotti-Krueger M, Ray T (1993). Cryopreservation of whole blood and isolated lymphocytes for B-cell immortalization. J. Tissue Culture Methods.

[87] Sie L, Loong S, Tan EK (2009). Utility of lymphoblastoid cell lines. J. Neurosci. Res..

[88] Coore HG, Denton RM, Martin BR, Randle PJ (1971). Regulation of adipose tissue pyruvate dehydrogenase by insulin and other hormones. Biochem. J..

[89] Kriks S (2011). Dopamine neurons derived from human ES cells efficiently engraft in animal models of Parkinson’s disease. Nature.

[90] Mazzulli JR, Zunke F, Isacson O, Studer L, Krainc D (2016). alpha-Synuclein-induced lysosomal dysfunction occurs through disruptions in protein trafficking in human midbrain synucleinopathy models. Proc. Natl Acad. Sci. USA.

[91] Fazzini F (2021). Association of mitochondrial DNA copy number with metabolic syndrome and type 2 diabetes in 14 176 individuals. J. Intern. Med..

