# Supplementary material for: Molecular phenotypes of mitochondrial dysfunction in clinically non-manifesting heterozygous PRKN variant carriers
Source: NPJ Parkinsons Dis. 2023 Apr 18;9:65. doi: 10.1038/s41531-023-00499-9 (PMC10113363; doi:10.1038/s41531-023-00499-9)
Supplement: Supplementary file 1 — Supplementary Information [file 41531_2023_499_MOESM1_ESM.pdf]

SUPPLEMENTARY INFORMATION

Supplementary Table 1. Individuals, for which LCLs and hiPSC-derived neurons were generated

ID	*PRKN mutation*	*PD status*	*LCLs*	*hiPSC-derived neurons*	*SN hyperechogenicity*	*Subtle motor signs (UPDRS-III)*	*Hypomimia*
PRKN+/PD- 1	heterozygous exon 7 deletion	unaffected	√	√	yes	yes	+
PRKN+/PD- 2	heterozygous exon 7 deletion	unaffected	√	√	yes	yes	+
PRKN+/PD- 3	heterozygous exon 7 deletion	unaffected	√		no	no	
PRKN+/PD- 4	heterozygous exon 7 deletion	unaffected	√		no	yes	+
PRKN+/PD- 5	heterozygous exon 7 deletion	unaffected	√		yes	no	
PRKN+/PD- 6	heterozygous exon 7 deletion	unaffected	√		no	no	
PRKN+/PD- 7	heterozygous exon 7 deletion	unaffected	√		no	no	
PRKN+/PD- 8	heterozygous exon 7 deletion	unaffected	√		yes	no	
PRKN+/PD- 9	heterozygous exon 7 deletion	unaffected	√		yes	no	
PRKN+/PD- 10	heterozygous exon 7 deletion	unaffected	√		yes	no	
PRKN+/PD- 11	heterozygous exon 7 deletion	unaffected	√		yes	yes	+
Control 1[1]	/	/	/	√	n.a.	n.a.	n.a.
Control 2	/	/	√	√	no	no	
Control 3	/	/		√	yes	no	
Control 4	/	/	√		no	no	
Control 5	/	/	√		yes	no	
Control 6	/	/	√		no	no	
Control 7	/	/	√		yes	yes	+
Control 8	/	/	√		no	no	
Control 9	/	/	√		yes	yes	+
Control 10	/	/	√		no	no	
Control 11	/	/	√		yes	no	
PRKN++/PD+1 [2]	exon 9 homozygous delA (p.Val324AlafsTer111 - rs1562519380)	Affected		√	n.a.	n.a.	n.a.
PRKN++/PD+2 [2]	homozygous exon 3 deletion	Affected		√	n.a.	n.a.	n.a.

In the age range of 30-54 years at examination, there are 7 males, 6 females, and 7 mutation carriers; in the age range of 55-80 years at examination, there are 5 males, 4 females, and 6 mutation carriers

SN: *substantia nigra*; UPDRS-III: Unified Parkinson's Disease Rating Scale (UPDRS)-Part III; n.a.: not available

SN hyperechogenicity, subtle motor signs, and hypomimia are regarded as markers for prodromal PD [1,2]

[1] healthy control line established through the StemBANCC consortium (https://cells.ebisc.org/STBCi033-B/)

[2] biallelic *PRKN*-PD lines included in the analyses for comparison [3,4]

Supplementary Table 2. Respiratory parameters assessed in LCLs of heterozygous *PRKN* variant carriers (n=11) and controls (n=9)

Respiratory parameters	***controls (n = 11)***		***PRKN+/PD- (n = 9)***		
	mean	*median*	*mean*	*median*	*p-value*
Routine respiration (R)	2.87±0.40	2.87	3.19±0.28	3.05	**0.023**
Leak respiration (L)	0.88±0.13	0.90	0.95±0.16	0.98	0.382
Maximal respiratory capacity (E)	6.58±1.20	6.69	7.08±0.99	6.70	0.518
Residual Oxygen Consumption (ROX)					
Rotenone	0.18±0.04	0.17	0.20±0.03	0.20	0.305
Antimycin A	0.22±0.03	0.22	0.24±0.03	0.24	0.138
ATP Turnover (R-L)	1.99±0.39	1.93	2.25±0.30	2.17	0.159
Spare Respiratory Capacity (ET-R)	3.71±0.82	3.71	3.89±0.74	3.51	0.909

Values are given as mean ± s.d. and median. Statistical analysis was carried out by using the Mann-Whitney U test. Bold p-value indicates significance with an alpha level of 0.05.

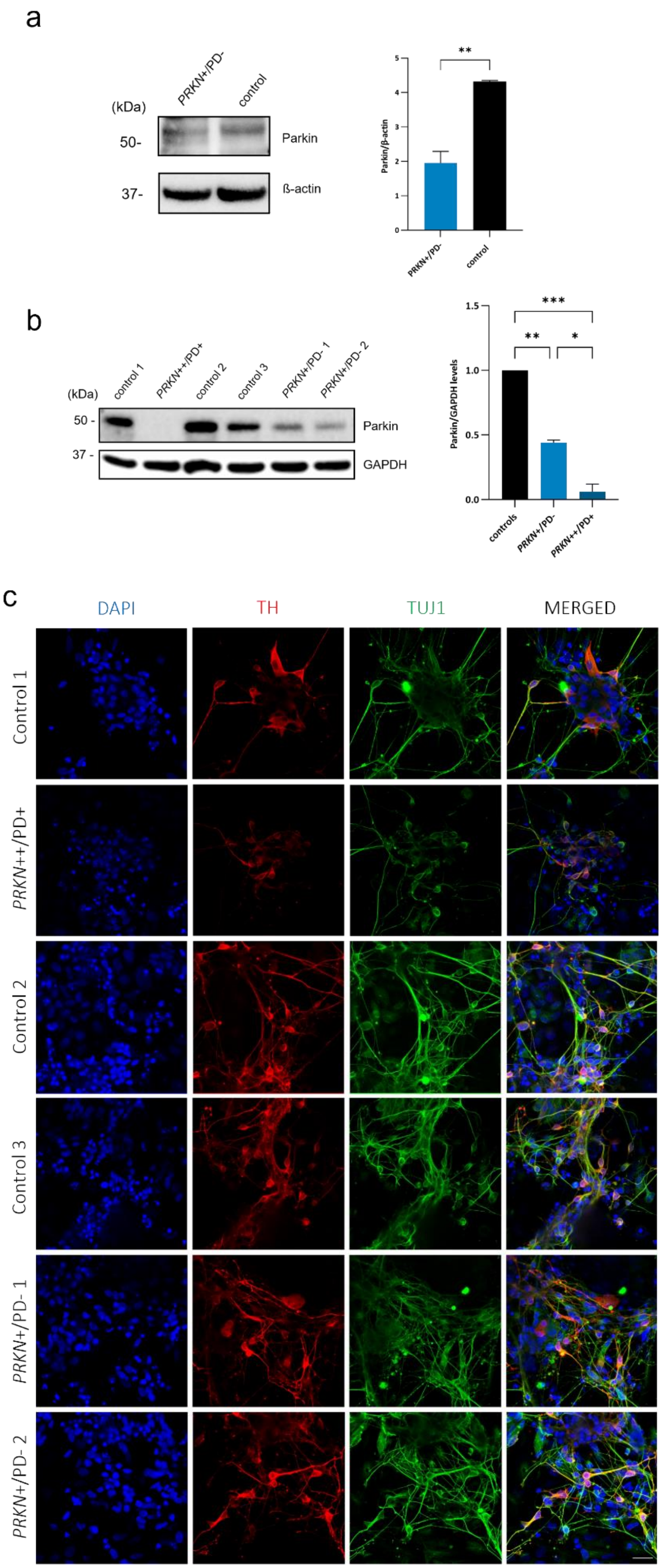
a
PRKN+/PD-
control
(kDa)
50-
37-
Parkin
ß-actin
**
Parkin/β-actin
0
1
2
3
4
5
b
control 1
PRKN++/PD+
control 2
control 3
PRKN+/PD- 1
PRKN+/PD- 2
(kDa)
50 -
37 -
Parkin
GAPDH
***
**
*
Parkin/GAPDH levels
0.0
0.5
1.0
1.5
controls
PRKN+/PD-
PRKN++/PD+
c
DAPI
TH
TUJ1
MERGED
Control 1
PRKN++/PD+
Control 2
Control 3
PRKN+/PD- 1
PRKN+/PD- 2

Supplementary Figure 1. Characterization of LCLs and hiPSC-derived neurons. (a) Western blot and densitometric analyses showing Parkin protein expression in LCLs of a control and a heterozygous *PRKN* variant carrier (*PRKN*+/PD-), n = 3. **(b)** Western blot and densitometric analyses showing Parkin protein expression in hiPSC-derived neurons of a PD line (*PRKN*++/PD+), controls, and heterozygous *PRKN* variant carriers (*PRKN*+/PD- 1, *PRKN*+/PD- 2), n = 2. Parkin levels were analyzed with an anti-Parkin antibody recognizing the C-terminus, GAPDH and β-actin were used as loading control. Molecular mass markers are in kilodaltons (kDa). Error bar represents the mean ± SEM. Significance levels were determined by using an Unpaired t test when comparing two groups, as well as one-way ANOVA and Tukey's *post hoc* test to correct for multiple comparisons. * p≤0.05, ** p≤0.01, *** p≤0.001. **(c)** Immunofluorescence staining of neuronal cultures derived from a PD line (*PRKN*++/PD+), controls, and heterozygous variant carriers (*PRKN*+/PD- 1, *PRKN*+/PD- 2), for neuron-specific TUJ1 (green), the DA marker TH (red), and nuclear DAPI (blue). Scale bar: 25 µm.

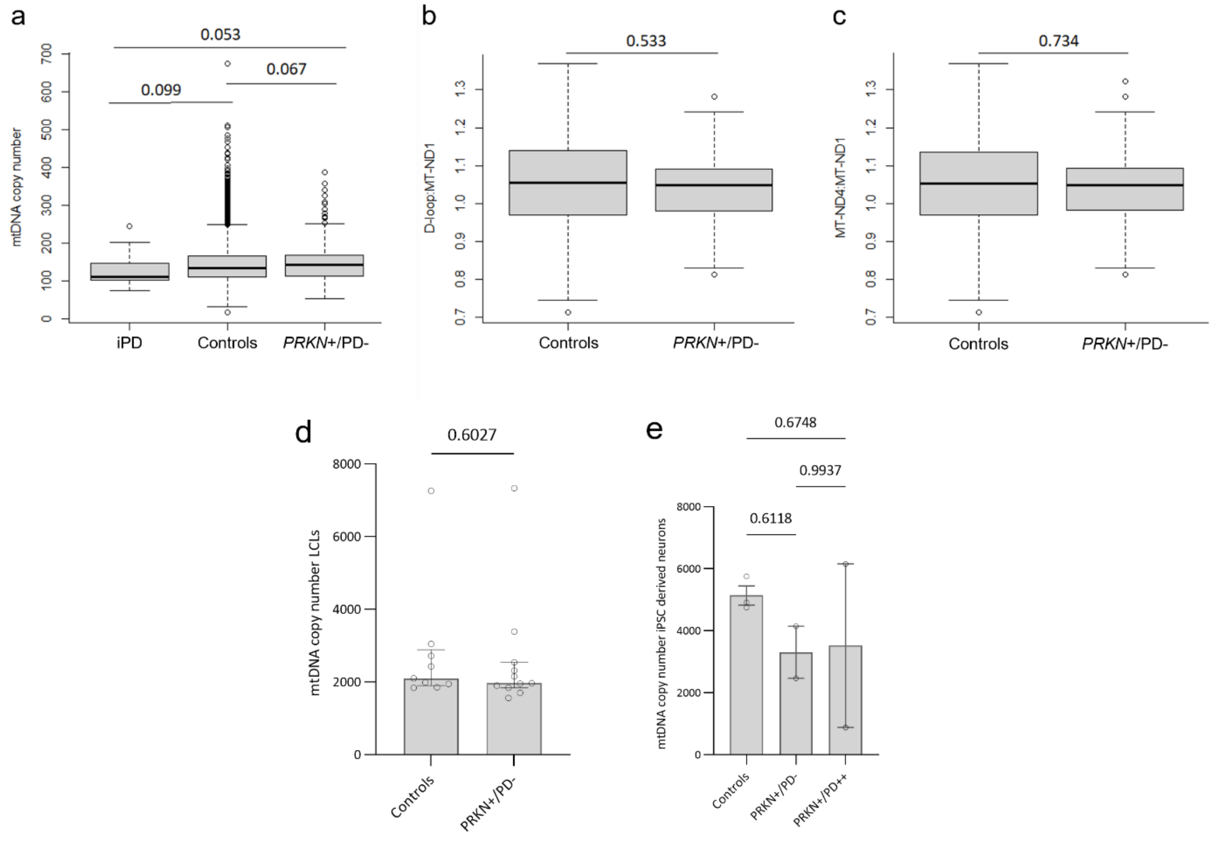

Supplementary Figure 2. mtDNA analyses in individuals carrying a heterozygous variant in *PRKN*. Real-time PCR quantification of the nuclear-encoded gene beta-2-microglobulin (*B2M*) and mtDNA sequences in the tRNA-Leu gene, the D-loop, the minor arc gene *MT-ND1* and the major arc gene *MT-ND4*. **(a)** tRNA-Leu:B2M ratio, indicating the amount of mtDNA copies in peripheral blood of individuals carrying a

heterozygous variant in *PRKN* (*PRKN*+/PD-) (n = 341), controls (n = 8,445), and iPD patients (n = 29). **(b)** D-loop:MT-ND1 ratio, indicating the amount of transcription-associated 7S DNA per mtDNA molecule in individuals carrying a heterozygous variant in *PRKN* (*PRKN*+/PD-) (n = 79), controls (n = 77). **(c)** MT-ND4:MT-ND1 ratio, indicating major arc deletions in individuals carrying a heterozygous variant in *PRKN* (*PRKN*+/PD-) (n = 79) and controls (n = 77). The horizontal line within the box indicates the median. **(d)** tRNA-Leu:B2M ratio, indicating the amount of mtDNA copies in LCLs of individuals carrying a heterozygous variant in *PRKN* (*PRKN*+/PD-) (n = 11) and controls (n = 9). **(e)** tRNA-Leu:B2M ratio, indicating the amount of mtDNA copies in iPSC-derived neurons of individuals carrying a heterozygous variant in *PRKN* (*PRKN*+/PD-) (n =2), controls (n = 3) and a *PRKN* PD patient (*PRKN*+/PD++) (n =2). Error bars represent the mean ± SEM. p values reported in the figures are the results of the Mann-Whitney test performed for each pair of groups within each mtDNA dataset and one-way ANOVA and Tukey's *post hoc* test to correct for multiple comparisons.

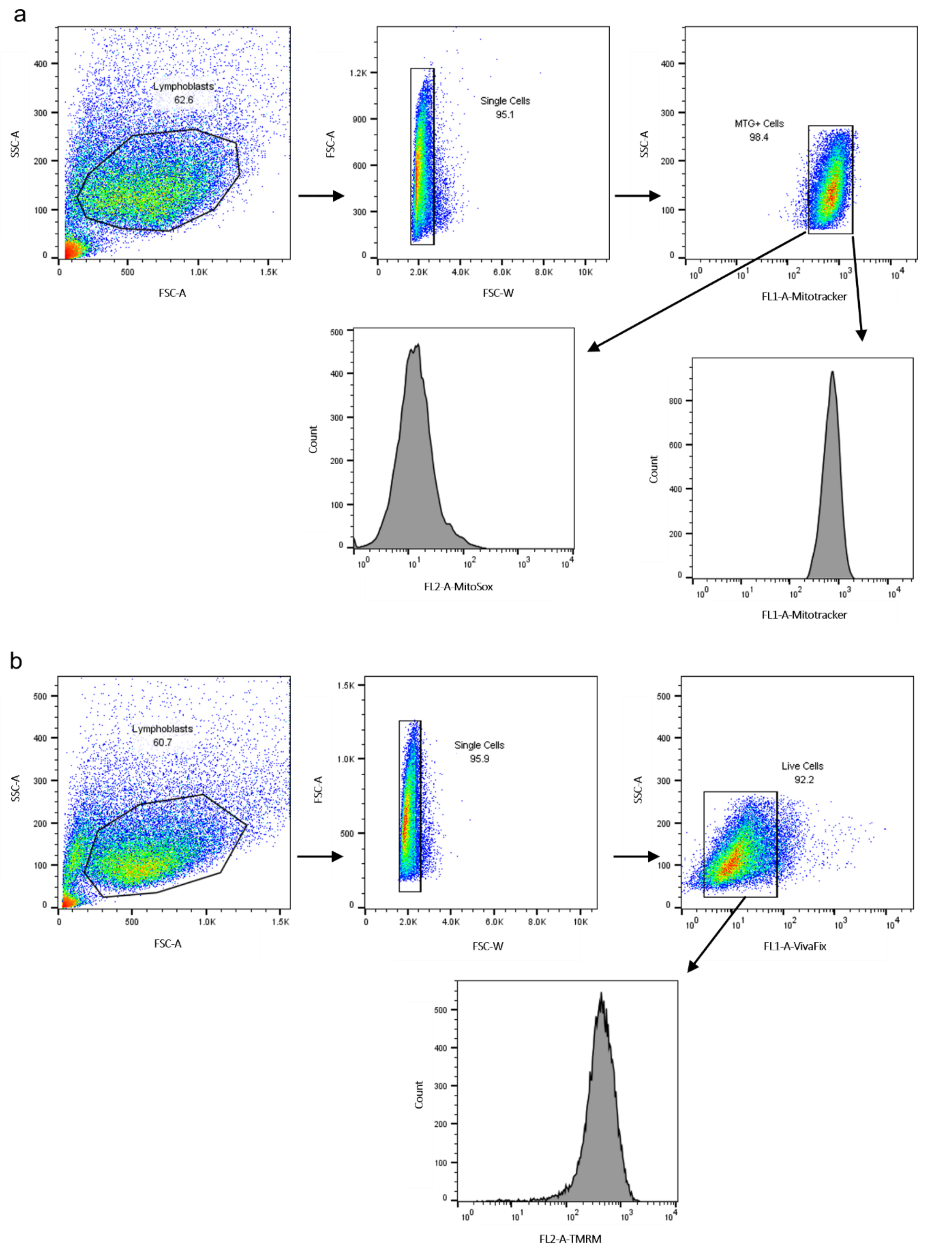

Supplementary Figure 3. Gating strategy for flow cytometry analysis in LCLs. Representative gating strategy plots of LCLs co-stained with **(a)** MitoTracker™ Green (MTG) and MitoSOX™ red to assess mROS

production (mitochondrial superoxide) and **(b)** co-stained with VivaFix™ and TMRM™ to determine mitochondrial membrane potential. The gating order is represented with black arrows and includes subsequent separation of LCLs (gated by forward (FSC-A) and sideward (SSC-A) scatter) and single cells (determined using FSC-A vs FSC-W). Next, MTG^{+} cells or live cells (VivaFix) were gated, and the mean value of MitoSOX red, MTG and TMRM was determined.

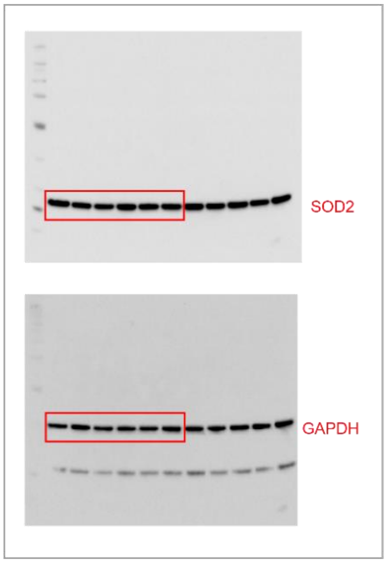

Uncropped western blots shown in Figure 4c

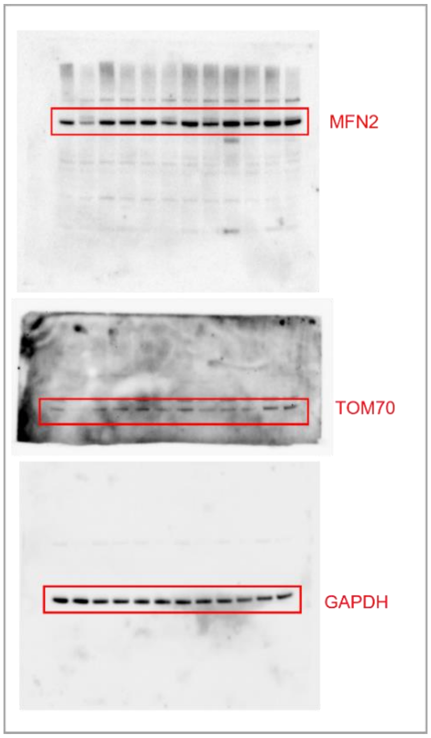

Uncropped western blots shown in Figure 6a

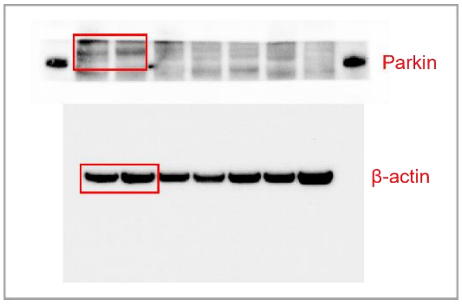

Uncropped western blots shown in Supplementary Figure 1a

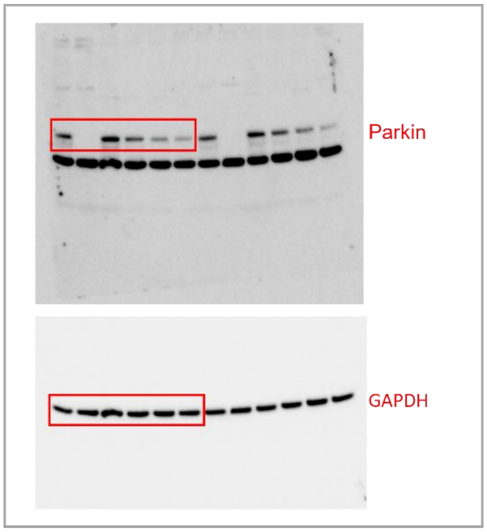

Uncropped western blots shown in Supplementary Figure 1b

References

1 Heinzel, S. *et al.* Update of the MDS research criteria for prodromal Parkinson's disease. *Mov Disord* **34**, 1464-1470 (2019). https://doi.org:10.1002/mds.27802

2 Fereshtehnejad, S. M. *et al.* Evolution of prodromal Parkinson's disease and dementia with Lewy bodies: a prospective study. *Brain* **142**, 2051-2067 (2019). https://doi.org:10.1093/brain/awz111

3 Zanon, A. *et al.* SLP-2 interacts with Parkin in mitochondria and prevents mitochondrial dysfunction in Parkin-deficient human iPSC-derived neurons and Drosophila. *Hum Mol Genet* **26**, 2412-2425 (2017). https://doi.org:10.1093/hmg/ddx132

4 Zanon, A. *et al.* Generation of an induced pluripotent stem cell line (EURACi005-A) from a Parkinson's disease patient carrying a homozygous exon 3 deletion in the *PRKN*gene. *Stem Cell Res* **41**, 101624 (2019). https://doi.org:10.1016/j.scr.2019.101624
